# A Mass Spectrometry-Based Profiling of Interactomes of Viral DDB1- and Cullin Ubiquitin Ligase-Binding Proteins Reveals NF-κB Inhibitory Activity of the HIV-2-Encoded Vpx

**DOI:** 10.3389/fimmu.2018.02978

**Published:** 2018-12-19

**Authors:** Christine D. Landsberg, Dominik A. Megger, Dominik Hotter, Meike U. Rückborn, Mareike Eilbrecht, Jassin Rashidi-Alavijeh, Sebastian Howe, Stefan Heinrichs, Daniel Sauter, Barbara Sitek, Vu Thuy Khanh Le-Trilling, Mirko Trilling

**Affiliations:** ^1^Institute for Virology, University Hospital Essen, University of Duisburg-Essen, Essen, Germany; ^2^Medical Proteome-Center, Ruhr-University Bochum, Bochum, Germany; ^3^Institute of Molecular Virology, Ulm University Medical Center, Ulm, Germany; ^4^Institute for Transfusion Medicine, University Hospital Essen, University of Duisburg-Essen, Essen, Germany

**Keywords:** interaction partner, mass spectrometry (MS), human immunodeficiency virus (HIV), hepatitis B virus (HBV), cytomegalovirus, NF-κB, interferon, DNA damage-binding protein (DDB1)

## Abstract

Viruses and hosts are situated in a molecular arms race. To avoid morbidity and mortality, hosts evolved antiviral restriction factors. These restriction factors exert selection pressure on the viruses and drive viral evolution toward increasingly efficient immune antagonists. Numerous viruses exploit cellular DNA damage-binding protein 1 (DDB1)-containing Cullin RocA ubiquitin ligases (CRLs) to induce the ubiquitination and subsequent proteasomal degradation of antiviral factors expressed by their hosts. To establish a comprehensive understanding of the underlying protein interaction networks, we performed immuno-affinity precipitations for a panel of DDB1-interacting proteins derived from viruses such as mouse cytomegalovirus (MCMV, Murid herpesvirus [MuHV] 1), rat cytomegalovirus Maastricht MuHV2, rat cytomegalovirus English MuHV8, human cytomegalovirus (HCMV), hepatitis B virus (HBV), and human immunodeficiency virus (HIV). Cellular interaction partners were identified and quantified by mass spectrometry (MS) and validated by classical biochemistry. The comparative approach enabled us to separate unspecific interactions from specific binding partners and revealed remarkable differences in the strength of interaction with DDB1. Our analysis confirmed several previously described interactions like the interaction of the MCMV-encoded interferon antagonist pM27 with STAT2. We extended known interactions to paralogous proteins like the interaction of the HBV-encoded HBx with different Spindlin proteins and documented interactions for the first time, which explain functional data like the interaction of the HIV-2-encoded Vpr with Bax. Additionally, several novel interactions were identified, such as the association of the HIV-2-encoded Vpx with the transcription factor RelA (also called p65). For the latter interaction, we documented a functional relevance in antagonizing NF-κB-driven gene expression. The mutation of the DDB1 binding interface of Vpx significantly impaired NF-κB inhibition, indicating that Vpx counteracts NF-κB signaling by a DDB1- and CRL-dependent mechanism. In summary, our findings improve the understanding of how viral pathogens hijack cellular DDB1 and CRLs to ensure efficient replication despite the expression of host restriction factors.

## Introduction

In response to viral infections, host cells have evolved a plethora of so-called restriction factors that inhibit specific steps of the viral replication cycle, thereby limiting the damage caused by infections. Several of these restriction factors are encoded by interferon (IFN)-stimulated genes (ISG) (1, 2). The antiviral activity elicited by restriction factors in turn exerts strong selection pressure on viruses, which have evolved sophisticated evasion and counteraction strategies (3).

One irreversible mechanism by which viral accessory proteins counteract restriction factors is the induction of their proteolytic degradation. To achieve this, viruses frequently exploit cellular pathways of protein degradation such as the proteasome. Although several pathways may target proteins for proteasomal degradation, ubiquitination is considered the most important one (4). Ubiquitin (Ub) is a small protein that is covalently linked to proteins acting as a molecular tag to mediate the recognition by the proteasome (5). Viruses inducing the proteasomal degradation of host restriction factors via ubiquitination (6, 7) often lose their replication capacity, if the Ub conjugation machinery or the proteasome is inactivated (8–12). However, an inhibition of the proteasome alters the abundance of more than 80% of all cellular proteins (13). Not surprisingly, such regimes are associated with severe toxicity limiting their application as antiviral drugs—despite their FDA approval as tumor drugs (e.g., Bortezomib). A specific inhibition of individual Ub ligases might limit side effects to levels tolerable in the context of antiviral therapies.

Cytomegaloviruses inhibit IFN-induced Jak-STAT signaling (14–20). Cytomegaloviruses exploit DNA damage-binding protein 1 (DDB1) and Cullin RocA ubiquitin ligases (CRLs) to antagonize IFN signaling (16, 21). In uninfected cells, DDB1 fulfills several cellular functions including nucleotide excision DNA repair (22). Consistent with the exploitation of DDB1, the CRL inhibitory drug MLN4924 (also called Pevonedistat) elicits potent antiviral activity against cytomegaloviruses and several other viruses (21). This is in agreement with the fact that several viral accessory proteins utilize DDB1 and/or CRLs to induce proteasomal degradation of host restriction factors: HIV-1 and HIV-2 encode Vpr, which interacts via VprBP with DDB1 and CRLs and influences processes such as the cell cycle by destabilizing host proteins (11, 12, 23–30). HIV-2 additionally expresses Vpx, which exploits DDB1 and CRLs, also via VprBP, to induce the degradation of SAMHD1 enabling replication in myeloid and dendritic cells (31–37). HBx and WHx, derived from HBV and the Woodchuck hepatitis virus (WHV), respectively, bind DDB1 and CRLs to induce the degradation of restriction factors such as SMC5/6 (38–42).

We applied immunoprecipitation (IP) coupled to mass spectrometry (MS) to establish a comprehensive and quantitative understanding of the complexes assembled by viral DDB1/CRL-interacting proteins and their targets.

## Materials and Methods

### Cells, Transfection, Infection

Human embryonic kidney (HEK) 293T (ATCC CRL-11268) and mouse NIH3T3 (ATCC CRL-1658) cells obtained from the American Type Culture Collection were used for transfection and infection experiments, respectively. Primary mouse fibroblasts [mouse embryonic fibroblasts [MEF] and mouse newborn cells [MNC]] were isolated from C57BL/6 and BALB/c embryos and newborn mice using protocols described in Le-Trilling and Trilling (43). These animal experiments were approved by local authorities and the corresponding ethics committee (permit number 84-02.04.2014.A390; name of the committee: the Ministry for Environment, Agriculture, Conservation and Consumer Protection of the State of North Rhine-Westphalia in Düsseldorf, Germany; address: LANUV-section 81, Recklinghausen, Germany). Immortalized mouse fibroblasts had been generated from primary C57BL/6 and BALB/c MEF by crisis immortalization (44). All cells were grown in Dulbecco's modified Eagle medium (DMEM) supplemented with 10% (v/v) fetal bovine serum, 100 μg/ml streptomycin, 100 U/ml penicillin, and 2 mM glutamine. Cell culture media and supplements were obtained from Gibco/Life technologies. The transfection of HEK293T cells was performed by the use of polyethylenimine hydrochloride (PEI, Sigma) at a concentration of 3 μg PEI/μg plasmid DNA. For the infection experiment, recombinant MCMV mutants expressing pM27-HA, pR27-HA, pUL27-HA, or eGFP were used. These mutants were constructed by flp-mediated recombination of an frt-site-flanked fragment—encompassing the human EF1 promoter in front of the respective gene—into a recombinant MCMV bacterial artificial chromosome (BAC) which already harbored an frt-site instead of the M27 sequence. Recombinant MCMVs were reconstituted by transfection of BAC DNA (Superfect, Qiagen) into permissive fibroblasts. For the preparation of MCMV stocks, immortalized MEF were used. Viral titers were determined by standard plaque titration on primary MEF or MNC (43). All infections and virus titrations were done with centrifugal enhancement (800 g for 30 min).

### Plasmids

The following expression constructs were used for the IP experiments: pIRES2-EGFP_Intron (BD Biosciences Clontech vector; intron sequence inserted into NheI site in front of the multiple cloning site), pIRES2-EGFP-M27-Flag [described in Trilling et al. (16)], pIRES2-EGFP-M27-HA, pIRES2-EGFP-E27-HA, pIRES2-EGFP-R27-HA, pIRES2-EGFP-UL27-HA, pIRES2-EGFP-Intron-HBx-HA, pIRES2-EGFP-WHx7-HA, pIRES2-EGFP-WHx8-HA, pcDNA-UL42-HA, pcDNA-Vpx-Flag [described in Lim et al. (45)], pcDNA-Vpr-Flag [described in Fregoso et al. (46)], pcDNA-Flag-DDB1 [received from Addgene, described in Hu et al. (47)], and PMZ3F-STAT2-SPA. M27 with a C-terminal HA epitope tag was cloned using the primers KL-M27-1 GAGGGATCCGCCTCTTCGAGGAG and KL-M27-HA2 GAGGGATCCTCAAGCGTAATCTGGAACATCGTATGGGTACACCCGCTCCACCACAAACTC to generate a PCR product which was cloned into the BamHI-cleaved pIRES2-EGFP-M27-Flag to exchange the epitope tag. Plasmids containing the coding sequence of the pM27-homologous protein pE27 of the *Murid herpesvirus 8* (“RCMV England”) were kindly provided by Sebastian Voigt, Robert-Koch-Institute Berlin and Charité Berlin. The E27 CDS fused to a C-terminal HA epitope tag was subcloned into pIRES2-EGFP by use of the NheI and EcoRI restriction sites. The CDS of pR27 of the Murid herpesvirus 2 (“RCMV Maastricht”) with a C-terminal HA epitope tag was ordered from GeneArt gene synthesis and subcloned into pIRES2-EGFP by NheI and HindIII sites. UL27 of HCMV was cloned by PCR amplification of the CDS using the primers KL-UL27-1 CGGCTAGCATGAACCCCGTGGATCAGCCG and KL-UL27-HA-2 CGGAATTCTCAAGCGTAATCTGGAACATCGTATGGGTATGTGGCGTGACCTCCGACCTC containing restriction sites and a C-terminal HA epitope tag. PCR products were cleaved with NheI and EcoRI and cloned into pIRES-EGFP2. Templates for the PCR amplification of HBx of HBV and the homologous x proteins (termed WHx7 and WHx8) of WHV were kindly provided by Mengji Lu, University of Duisburg-Essen. For the cloning into the expression vectors, primers containing restriction sites and a C-terminal HA epitope tag were used: JR-HBx-1 CGGCTAGCATGGCTGCTAGGCTGTGCTG and JR-HBx-HA-2 CGGAATTCTTAAGCGTAATCTGGAACATCGTATGGGTAGGCAGAGGTGAAAAAGTTGCATG for HBx and JR-WHx-1 CGGCTAGCATGGCTGCTCGCCTGTGTTG and JR-WHx-HA-2: CGGAATTCTTAAGCGTAATCTGGAACATCGTATGGGTACAGAAGTCGCATGCATTTATGCC for WHx7 and WHx8. The following primers were used for the cloning of the N-terminal HA tagged HBx: HA-HBx-1 CGGCTAGCATGTACCCATACGATGTTCCAGATTACGCTGCTGCTAGGCTGTGCTGCC and HA-HBx-2 CGGAATTCTTAGGCAGAGGTGAAAAAGTTGCATG. PCR products were cleaved with NheI and EcoRI and cloned into pIRES-EGFP2_Intron in the case of HBx and pIRES-EGFP2 in the case of WHx7 and WHx8. pUL42-HA was expressed from pcDNA3.1 (Invitrogen) and was cloned using the following primers: UL42-HA for ATCGTCAAGCTTATGGAGCCCACGCCGATGCTC and UL42-HA rev GACGATGAATTCTTACGCGTAATCTGGAACATCGTATGGGTACCCCGATGATGCTTGCGT. For the cloning of pMZ3F-STAT2-SPA, the primers STAT2-SPA for CCTCGAGA TGGCGCAGTGGGAAATGCTGC and STAT2-SPA rev GGCGGCCGCGAAGTCAGAAGGCATCAAGGGTC were used to generate a PCR product which was cleaved with XhoI and NotI and inserted into the pMZ3F vector (48), which was kindly provided by the lab of Jack Greenblatt, University of Toronto.

Vpr and Vpx, both encoded by HIV-2 (TaxID 11709) Rod-9, expression plasmids were generated by Michael Emerman, Fred Hutchinson Cancer Research Center, and provided to us by Hanna-Mari Baldauf, University Hospital Frankfurt.

HIV-2 Rod10 vpx (49) and HIV-1 M CH106 vpu (50) were cloned via XbaI and MluI restriction sites into bicistronic pCG vectors co-expressing eGFP via an internal ribosome entry site (IRES). The NF-κB firefly luciferase reporter construct as well as the expression plasmids for p65, a constitutively active mutant of IKKβ (S177E, S181E) and dominant negative mutants of IKKα and IKKβ were described in Sauter et al. (50) and kindly provided by Bernd Baumann, Ulm University. The pTAL luciferase vector used for normalization was generated by replacing the firefly luciferase gene of a reporter construct purchased from Clontech (# 631909) with a *gaussia* luciferase gene.

### Immunoprecipitation

HEK293T cells were washed three times with ice-cold PBS and subsequently lysed by incubation for 1 h on ice in lysis buffer (16). The indicated antibody was added to the supernatant and incubated overnight. Protein-G-sepharose was added and incubated for 1 h. Protein-G-sepharose-bound proteins were washed six times using lysis buffer containing 150–500 mM NaCl. Protein complexes were analyzed by silver-stained SDS-PAGE gels, immunoblotting, or mass spectrometry.

### Immunoblotting

Protein and IP samples were separated by classic SDS-PAGE, transferred to nitrocellulose membranes and probed with the following commercially available antibodies: rabbit α-Connexin-43 (Cell Signaling), mouse α-Itch (BD Transduction Laboratories), mouse α-HA (Sigma), rabbit α-HA (Sigma), rabbit α-DDB1 (Bethyl Laboratories Inc), rabbit α-Ncoa5 (Bethyl Laboratories Inc), rabbit α-Xpo7 (Proteintech), mouse α-Flag M2 (Sigma), or mouse α-p65 F6 (Santa Cruz Biotechnology). Xpo7 was also analyzed using an antibody which has been described previously (51) and which was generously provided by Dirk Görlich, Max-Planck Institut f. Biophysikalische Chemie in Göttingen, Germany. After incubation with the appropriate peroxidase-coupled secondary antibodies, signals were visualized with the ECL chemiluminescence system (Cell Signaling).

### MS-Based Analysis of Viral Interactomes

For in-gel digestions, 20 μl of each sample were applied to SDS-PAGE to collect the proteins in a single band for each sample. Protein bands were stained with Coomassie Brilliant Blue and subsequently digested with trypsin (SERVA) overnight at 37°C. Peptides were extracted using 50% (v/v) acetonitrile in 0.1% (v/v) trifluoroacetic acid (TFA) and sonicated twice on ice for 10 min. Extracts were dried by vacuum centrifugation and dissolved in 34 μl 0.1% (v/v) TFA. The peptide concentration was determined by quantitative amino acid analysis performed on an ACQUITY-UPLC equipped with AccQ Tag Ultra-UPLC column (Waters). For calibration, Pierce Amino Acid Standard (Thermo Scientific) was used.

Of each sample, 350 ng tryptic peptides were analyzed by LC-ESI-MS/MS on an Orbitrap Elite mass spectrometer online-coupled to an Ultimate 3000 RSLCnano system (both Thermo Scientific). After injection, peptides were trapped on a pre-column (Acclaim PepMap 100, 300 μm × 5 mm, C18, 5 μm, 100 Å) at a flow rate of 30 μL/min (0.1% trifluoroacetic acid). After 7 min, peptides were transferred to the analytical column (Acclaim PepMap RSLC, 75 μm × 50 cm, nano Viper, C18, 2 μm, 100 Å). A 98 min gradient of 5 to 40% of buffer B (0.1% formic acid, 84% acetonitrile) in buffer A (0.1% formic acid) was applied to elute the peptides from the analytical column (flow rate 400 nL/min, column oven temperature 60°C). Full MS spectra were acquired in the Orbitrap analyzer at a mass resolution of 60,000 (mass range 350–2000 m/z). Fragment mass spectra were acquired in data-dependent mode and recorded in the linear ion trap. The twenty most abundant precursor ions were selected for fragmentation using collision-induced dissociation (CID) with a normalized collision energy of 35% and an isolation width of 2.0 m/z.

Protein identification was conducted with Proteome Discoverer software (ver. 1.4.1.14, Thermo Fisher Scientific). Based on the human proteome file UP000005640 downloaded from Uniprot-KB (release 2015_05), a database was constructed containing 68,840 human protein sequences (UniProtKB/Swiss-Prot and UniProtKB/TrEMBL entries) and sequences of eGFP, HA-tagged proteins pM27, HBx, WHx7, WHx8, pR27, pE27, pUL27, pUL42 as well as Flag-tagged proteins Vpr and Vpx. For database searches, Sequest HT algorithm was used as implemented in the respective version of Proteome Discoverer software. Mass tolerance was set to 5 ppm for precursor ions and 0.4 Da for fragment ions. One tryptic miscleavage and variable modifications of methionine (oxidation) and cysteine (propionamide) were allowed. Peptide confidence was estimated using the Target Decoy PSM Validator function implemented in Proteome Discoverer. Peptides with a false discovery rate <1% were considered for analysis. Protein grouping function based on strict maximum parsimony principle was enabled in Proteome Discoverer.

Proteomics data have been deposited to the ProteomeXchange Consortium (http://proteomecentral.proteomexchange.org) (52) via the PRIDE partner repository (53) with the data identifier PXD007634 and DOI 10.6019/PXD007634. Files in the mzIdentML standard format were generated using ProCon—PROteomics CONversion tool (ver. 0.9.641) (54).

### NF-κB Promoter Assays

HEK293T cells were co-transfected with an NF-κB firefly luciferase reporter, a *Gaussia* luciferase construct under the control of a constitutively active pTAL promoter for normalization, and expression vectors for HIV-2 Rod10 Vpx, HIV-1 M CH106 Vpu or dominant negative mutants of IKKα or IKKβ. To activate NF-κB, an expression vector for p65/RelA or a constitutively active mutant of IKKβ was co-transfected, or cells were stimulated with TNFα for 24 h. Two days post-transfection, a dual luciferase assay was performed and the firefly luciferase signals were normalized to the corresponding *Gaussia* luciferase control values.

### Statistical Testing

A two-tailed and heteroscedastic (assuming different variances of the compared subpopulations) Student's *t*-Test was used to compare the indicated data sets.

## Results

### Viral DDB1-Binding Proteins Exhibit Distinct Co-precipitation Profiles

**Figure 1:**
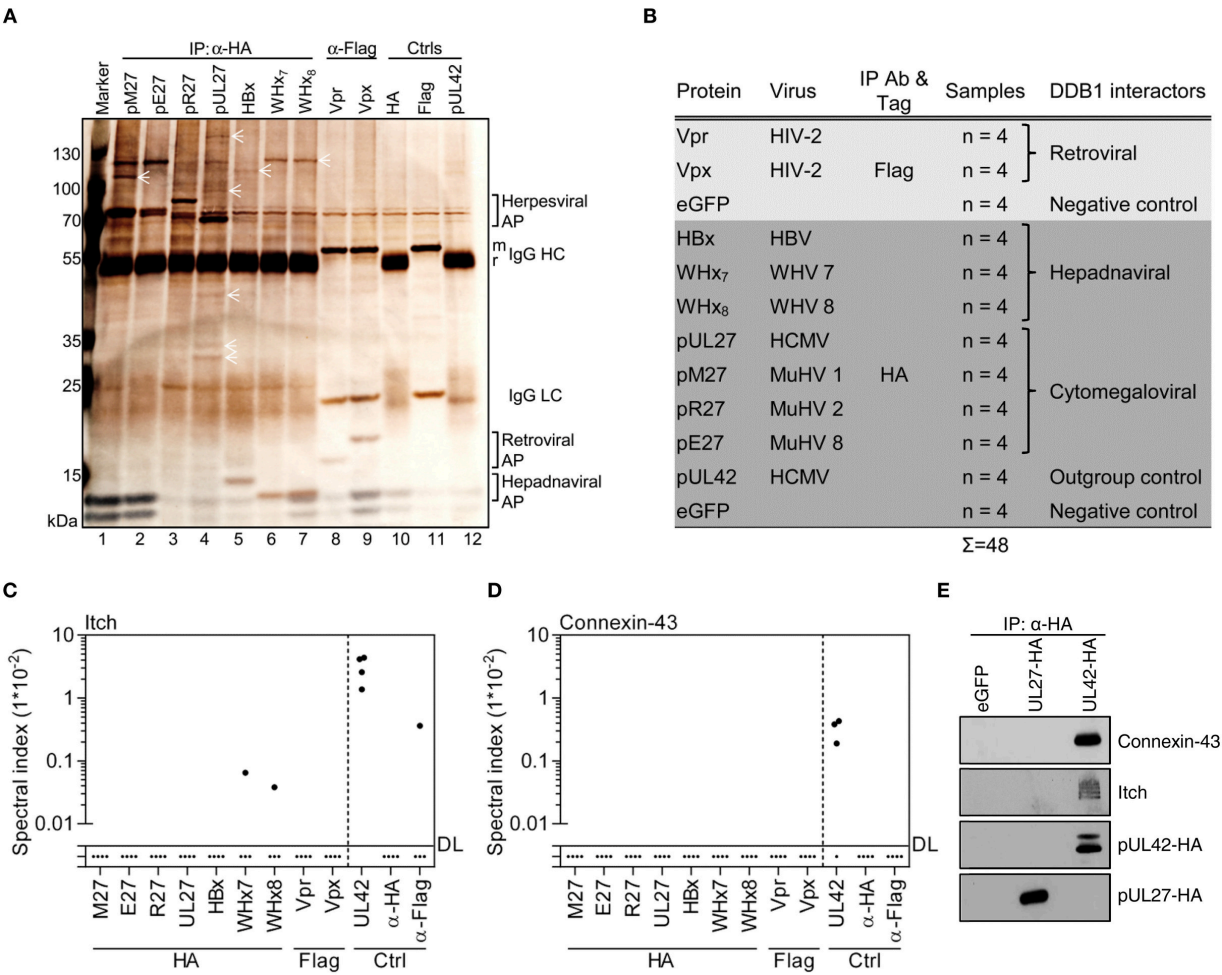
Viral DDB1-interacting proteins exhibit distinct co-precipitation profiles. **(A)** Accessory proteins implicated in the exploitation of DDB1 were expressed in HEK293T cells. Lysates were generated and subjected to IP analysis using the indicated antibodies. Retrieved proteins were visualized by silver staining of SDS gels. Symbols highlight proteins representing partially overlapping and specific interactions. Control IPs were performed using cells transfected with pUL42- and eGFP-expressing plasmids. **(B)** Overview of the experimental conditions for IP coupled to mass spectrometry (MS). The indicated proteins were analyzed by IP as described in **(A)** but instead of using silver stained gels, (co-)precipitated proteins were identified and quantified by MS in four biological replicates. Spectral indexes were calculated for Itch **(C)** and Connexin-43 **(D)**. **(E)** The indicated proteins were expressed in HEK293T cells. Lysates were subjected to IP using an HA-specific antibody and immunoblot analysis was performed for the indicated proteins.

Based on the finding that the MCMV-encoded accessory protein pM27 exploits Cul4A and DDB1 to induce ubiquitination and proteasomal degradation of STAT2, we studied pM27, its HCMV-derived homolog pUL27 as well as pE27 and pR27 of *Murid herpesvirus* (MuHV) 8 and MuHV2, respectively. We further included the DDB1-binding protein HBx of HBV and two WHV homologs derived from genotypes 7 and 8 termed WHx7 and WHx8. Vpr and Vpx encoded by HIV-2 were also included. GFP-expressing cells served as controls. The viral proteins harboring either an HA (pM27, pE27, pR27, pUL27, HBx, WHx7, and WHx8) or a Flag epitope tag (Vpr and Vpx) were expressed in transiently transfected HEK293T cells. After overnight incubation, cells were lysed and subjected to IP using HA- or Flag-specific antibodies, and protein G sepharose, as described in the Material and Methods section. In silver stained SDS-PAGE gels, pM27, pE27, and the WHx proteins interacted with a ~125 kDa protein supposed to be DDB1 (~127 kDa) (Figure 1A). Interestingly, no such precipitation was detected in the case of HBx, pUL27, or pR27. Additionally, patterns of specific as well as overlapping interactions became evident.

Since size alone is not a proper way to identify a protein, the immuno-purified complexes were subjected to MS to identify, quantify, and compare the interactomes of these viral accessory proteins (Figure 1B). As outlined in more detail below, this comprehensive analysis confirmed previously described interactions, extended known interactions to paralogous host and virus proteins, and identified numerous novel interaction partners.

### HCMV-Derived pUL42 Forms a Network of Proteins Harboring WW Domains or PPxY Motifs

In our analyses, we included pUL42 as outgroup control. This HCMV-encoded protein does not recruit DDB1 or CRLs, but interacts with the WW domain of the NEDD4-like Ub ligase Itch via its PPxY motif (55). In agreement with this, pUL42 interacted with Itch (Figure 1C) but not with DDB1 or CRL components in our experiments (Figure S1). Furthermore, pUL42 retrieved several additional members of the NEDD4 Ub ligase family (NEDD4, NEDD4L, WWP1, and WWP2), known binding partners of NEDD4 Ub ligases (e.g., Connexin-43), other proteins containing WW domains (e.g., YAP1, STXBP4, and BAG3), and their interaction partners such as VAMP-2 (Figure 1D and Figures S1, S2). Notably, Connexin-43 has previously been shown to be downregulated upon HCMV infection (56), and we confirmed its interaction with pUL42 using IP and immunoblotting (Figure 1E). Taken together, these data indicate that the HCMV-encoded pUL42 constitutes a suitable outgroup for our approach and reveal that it assembles a protein network most likely bridged through WW domains and corresponding PPxY motifs.

### The Interactome of pM27 Supports and Extends the Current Model of pM27 Function

**Figure 2:**
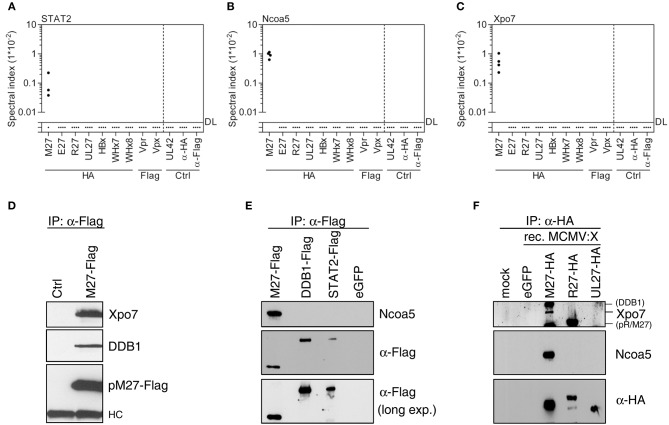
MCMV-encoded pM27 interacts with STAT2, Xpo7, and Ncoa5. Based on the MS data set (see Figure 1B), spectral indexes were calculated for STAT2 **(A)**, Ncoa5 **(B)**, and Xpo7 **(C)**. **(D)** NIH3T3 cells were infected (24 h; 5 PFU/cell) with recombinant VACV mutants expressing STAT2-HA (“ctrl”) or pM27-Flag [both described in Zimmermann et al. (14)] to validate the interaction between pM27 and Xpo7. **(E)** The indicated proteins were expressed in HEK293T cells. Lysates were subjected to IP and subsequent immunoblotting using the indicated antibodies. **(F)** NIH3T3 cells were infected (24 h; 3 PFU/cell) with recombinant MCMV mutants expressing the indicated cytomegaloviral HA-tagged proteins. The proteins were precipitated using an HA-specific antibody. (Co-) precipitated proteins were analyzed by immunoblotting using the indicated antibodies.

After definition of the outgroup, the interaction network of MCMV pM27 was assessed. As expected from previous work (16), pM27 efficiently retrieved STAT2 (Figure 2A), DDB1, and Cul4A (Figure S3). Additionally, several novel interaction partners, including Ncoa5 and Xpo7, were identified (Figures 2B,C and Figure S3). Ncoa5 is an interaction partner of nuclear receptors and exhibits co-activator and co-repressor functions (57, 58). Xpo7 regulates the import and export of several proteins to and from the nucleus (59). Since we had shown before that pM27 precipitates STAT2, DDB1, and Cul4A (14, 16) these herein confirmed interactions strengthen the reliability of our approach. As additional validation, the novel interactions were confirmed by IP and immunoblotting using constructs harboring Flag- instead of HA-epitopes (Figures 2D,E). To assess these interactions under infection conditions, we generated recombinant MCMV mutants expressing eGFP or HA epitope-tagged versions of pM27, pR27, or pUL27. In agreement with above experiments, Ncoa5 and Xpo7 exist in complexes with pM27 in MCMV-infected cells (Figure 2F), confirming that our MS approach reliably uncovers novel interaction partners of viral accessory proteins.

### DDB1-Interacting Viral Accessory Proteins Co-precipitate DDB1 With Distinct Efficiencies

**Figure 3:**
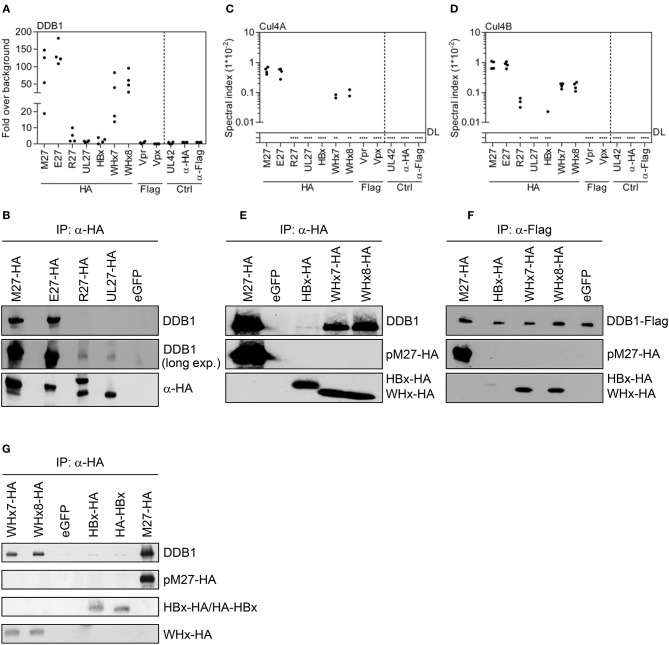
Viral DDB1-interacting accessory proteins co-precipitate DDB1 with distinct efficiencies. Based on the MS data set (see Figure 1B), spectral indexes were calculated. **(A)** The abundance of DDB1 was calculated as fold over background based on the spectral indexes. **(B)** The co-precipitation of DDB1 was further analyzed for the herpesviral DDB1-binding proteins by IP and subsequent immunoblotting. The spectral indexes for Cul4B and Cul4A are depicted in **(C,D)**, respectively. **(E)** The co-precipitation of DDB1 with the hepadnaviral DDB1-binding proteins was analyzed by IP and subsequent immunoblotting. **(F)** The DDB1 interaction was further analyzed by a reverse approach in which Flag-tagged DDB1 was expressed together with the viral accessory proteins. Lysates were generated, subjected to IP using an α-Flag antibody, and analyzed by immunoblotting.

The SDS-PAGE experiments already suggested differences regarding the strength of interaction between viral accessory proteins and DDB1. Consistently, quantitative MS revealed that pM27 retrieved DDB1 ~60-fold more efficiently than pUL27 (Figure 3A). The protein pE27 also strongly bound DDB1 (124.9 ± 27.6-fold over background), whereas the DDB1 interaction was clearly less pronounced in the case of pR27 (3.6 ± 3.4-fold over background) (Figure 3A). These differences were verified by IP and immunoblot analysis (Figure 3B). The differential interaction strengths between viral accessory proteins and DDB1 were also recapitulated on the level of Cul4A/B co-precipitation: pM27 and pE27, but not pUL27, efficiently co-precipitated Cul4A and Cul4B (Figures 3C,D). The protein pR27 precipitated Cul4B to a certain extent, albeit clearly less efficient as compared to pM27 and pE27 (Figure 3D).

For hepadnaviral HBx and WHx proteins, the interaction with DDB1 is well-described and even substantiated by co-crystallization of DDB1 with synthetic α-helical peptides corresponding to HBx or WHx (39). Consistent with our SDS-PAGE analysis, WHx7 and WHx8 co-precipitated DDB1 very efficiently (Figure 3A), whereas HBx only weakly interacted with DDB1 (2.0 ± 1.5-fold over background; found only in 3 of 4 replicates). WHx7 and WHx8 also precipitated Cul4A and Cul4B, whereas Cul4B was only found in a single HBx IP replicate and Cul4A was not precipitated by HBx at all (Figures 3C,D). These differences in DDB1 interaction were substantiated by IP and subsequent immunoblot analysis (Figure 3E) and a reverse approach, in which DDB1 was precipitated and HBx, WHx7, and WHx8 were detected (Figure 3F). To rule out that the C-terminal HA epitope impairs the interaction between HBx-HA and DDB1, we generated a HBx version harboring an N-terminal HA tag (HA-HBx). However, HA-HBx and HBx-HA exhibited comparable weak DDB1 interactions (Figure 3G). Given the wealth of information on HBx and DDB1 (38, 60–65), the rather weak interaction of HBx with DDB1 and Cul4A/B, especially in contrast to its homologs derived from WHV, was surprising and revealed highly diverse affinities of viral accessory proteins for DDB1, Cul4A, and Cul4B.

### Differential Interaction Strengths of Viral Accessory Proteins With DDB1, Cul4A, and Cul4B Suggest a Preference of DDB1 for Cul4B

Interactions of the viral accessory proteins with Cul4A or Cul4B were only detected when DDB1 was also precipitated. The strength of interaction with DDB1 correlated positively with the retrieval of Cul4A and Cul4B (Figures S4A,B), suggesting that the viral proteins primarily recognize DDB1 and co-precipitate Cul4A and Cul4B indirectly via their interaction with DDB1. This is consistent with crystallization and functional data, which show that DDB1 mutations destabilizing the DDB1-Cul4A/B interface abrogate DDB1-dependent effects elicited by HBx (39). As expected, the co-precipitations of Cul4A and Cul4B with the viral accessory proteins showed a highly significant (*p* < 0.0001) positive correlation (Figure S4C). The functions of Cul4A and Cul4B are largely overlapping, but discrete functions do also exist (66). DDB1 binds the N-termini of Cul4A and Cul4B. Interestingly, the N-terminus of Cul4B is 149 amino acids longer than that of Cul4A and contains a nuclear localization signal. In all cases and supported by the slope of the linear correlation (0.58 ± 0.03), Cul4B was co-precipitated more efficiently than Cul4A (Figure S4C). Taking into account that Cul4A and Cul4B co-precipitated indirectly via DDB1, this argues in favor of a preference of DDB1 for Cul4B as compared to Cul4A.

### Confirmation and Extension of Known Interactions of Viral Accessory Proteins

**Figure 4:**
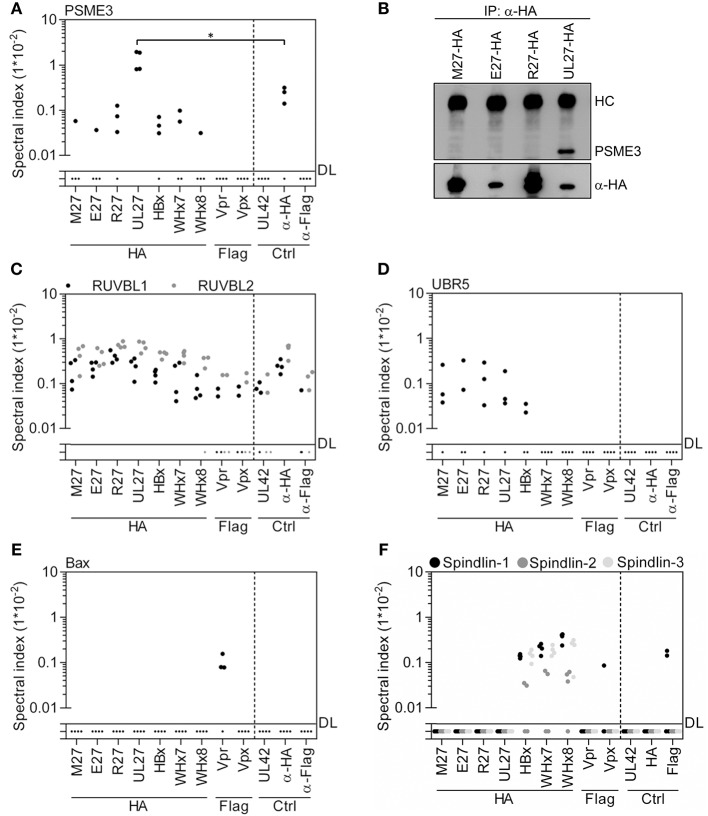
Exclusive and global interaction partners of viral accessory proteins. Based on the MS data set (see Figure 1B), spectral indexes were calculated. **(A)** pUL27 precipitated with PSME3. Since PSME3 precipitated to certain extend in the control conditions, the statistical significance was calculated. A level of significance corresponding to *p* < 0.05 is indicated by “*”. **(B)** The interaction was confirmed by IP and subsequent immunoblotting. **(C)** Unspecific precipitation of RUVBL1 and RUVBL2 with viral and control proteins is shown. **(D)** Herpesviral accessory proteins interact with UBR5. **(E)** HIV-2 Vpr co-precipitated Bax. **(F)** Precipitation data for Spindlin-1, -2, and -3 are depicted.

Reitsma et al. determined the interactome of pUL27 and presented a list of 27 precipitated proteins including PSME3, RUVBL2, RUVBL1, and UBR5 (67). Twenty-one of these were also quantified in our study, with 19 precipitating with pUL27. Twelve had spectral indexes above the background precipitation, of which Proteasome activator complex subunit 3 (PSME3; also called PA28γ), a part of the immune proteasome, was the most prevalent (Figure 4A and Figure S5). Although PSME3 precipitated to a certain extent in the HA control conditions, the precipitation by pUL27 was 6.89-fold and significantly (*p* < 0.03) above background. Immunoblot experiments confirmed the specific interaction of PSME3 with pUL27 (Figure 4B). RUVBL1 and RUVBL2 appeared in the precipitates of most viral accessory proteins and the control settings (Figure 4C), suggesting an unspecific retrieval and highlighting the advantage of such comparative experimental designs. DDB1 assembles two different Ub ligase complexes either containing Cul4A/B or the non-CRL Ub ligase UBR5 (28). Several herpesviral proteins interacted with UBR5, confirming the described interaction with pUL27 and extending it to its homologs pM27, pE27, and pR27 (Figure 4D). The fact that recruitment of UBR5 is evolutionarily conserved among different herpesviruses suggests a selection advantage of this interaction for viral replication *in vivo*.

Consistent with previous studies (68, 69), HIV-2-derived Vpr co-precipitated VprBP (Figure S6). Furthermore, we found an interaction between Bax and Vpr (Figure 4E) which is in line with published functional data but has to our knowledge not been experimentally validated before.

Recently, it was reported that HBx interacts with Spindlin-1 to counteract its inhibition of viral transcription (70). In addition to Spindlin-1, HBx, WHx7, and WHx8 also retrieved its homologs Spindlin-2 and Spindlin-3 (Figure 4F). Taken together, our analysis confirmed and significantly extended known or anticipated interactions of viral accessory proteins.

### Global Comparison of the Interactomes of Viral DDB1-Interacting Proteins Highlights Overlapping Mechanisms

**Figure 5:**
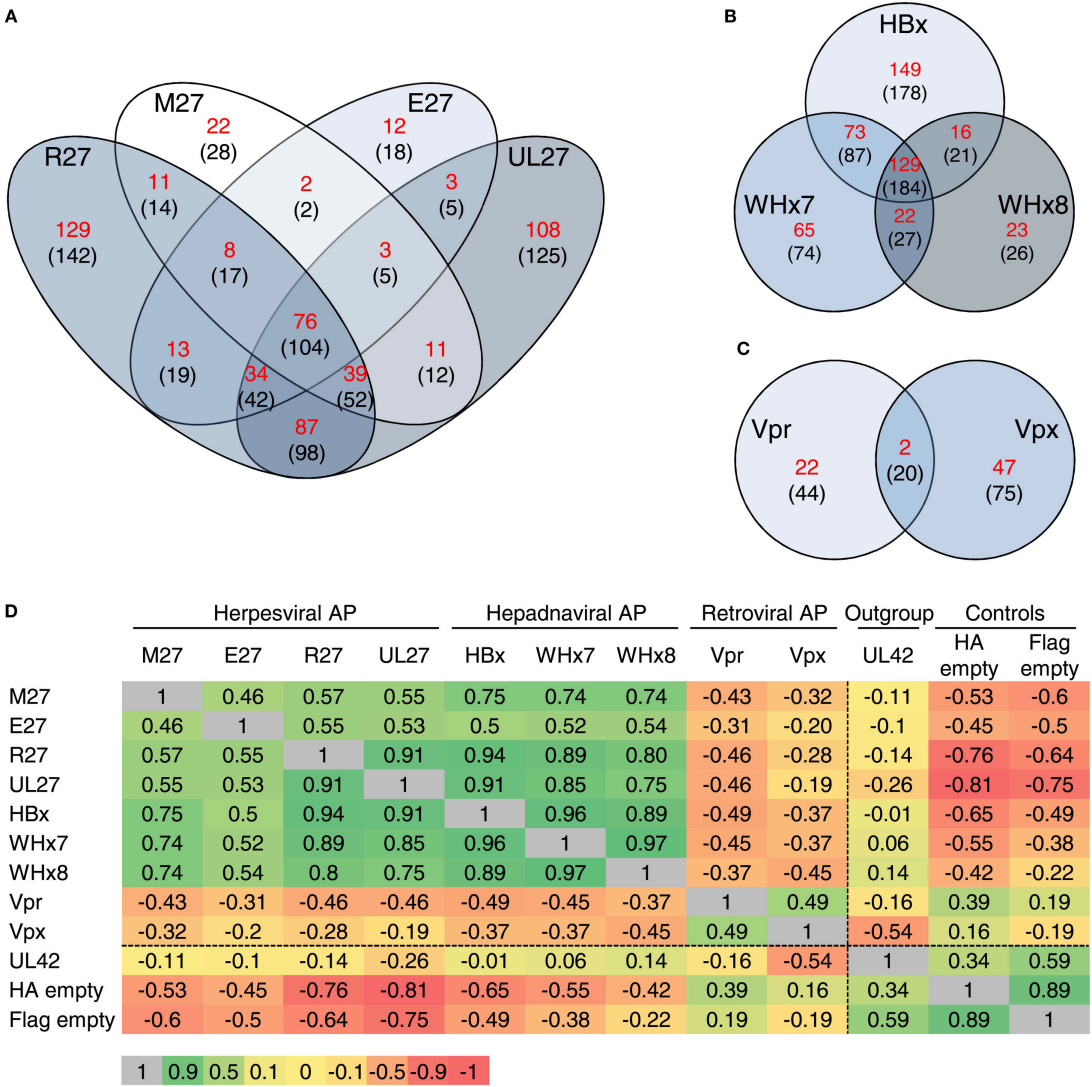
Global comparison of the interactomes of viral DDB1-interacting proteins. Venn diagrams depict the number of interactions shared between the indicated viral proteins. Interaction partners found in at least 2 of 4 replicates were included in the analysis. The calculation was performed for proteins, which were not co-precipitated in any of the control settings (numbers depicted in red) and for all proteins which were at least 2-fold enriched compared to the controls (numbers shown in brackets). The analysis was performed for the herpesviral **(A)**, hepadnaviral **(B)**, and retroviral **(C)** accessory proteins. **(D)** For a global comparison, Pearson's correlation coefficients for the entire interactome were calculated and depicted as a heat map including the correlation coefficients. Green color indicates a positive correlation between the interactomes whereas red color depicts an inverse correlation.

For a comparison of overlapping and specific interactions of viral accessory proteins with cellular factors according to their phylogeny, we restricted the analysis to interacting proteins found in at least two out of four replicates. Additionally, we compared interactors enriched at least 2-fold above the background (Figures 5A–C; numbers in brackets) as well as specific interactors not found in any of the control settings (see red numbers). Tables summarizing the interaction partners of the individual proteins or taxonomic as well as functional groups of viral accessory proteins can be found in the supplementary data (Supplementary Tables 1–10). For a systematic overview, we performed a global analysis based on individual correlation coefficients calculated on the spectral indexes of all interactors (Figure 5D). Consistent with the existence of a so-called “CRAPome” (71), HA and Flag control IPs correlated positively with each other. Neither the controls nor the outgroup correlated positively with the interactomes of the DDB1/CRL-interacting viral proteins, highlighting the specificity of our approach. The interactomes of the retroviral proteins Vpr and Vpx correlated positively with each other and showed an inverse correlation with the interactomes of the analyzed non-retroviral proteins. The interactomes of the hepadnaviral proteins HBx, WHx7, and WHx8 correlated strongly with each other. This suggests that the global interactomes reflect the phylogenetic relationship of homologous proteins. We also observed a positive correlation between unrelated proteins such as in the case of herpesviral and hepadnaviral proteins (e.g., pM27 and pE27 with WHx) which we interpret as a footprint within the global interactome based on similar functions.

### HIV-2 Vpx Co-precipitates p65/RelA and Inhibits NF-κB-Driven Gene Expression

**Figure 6:**
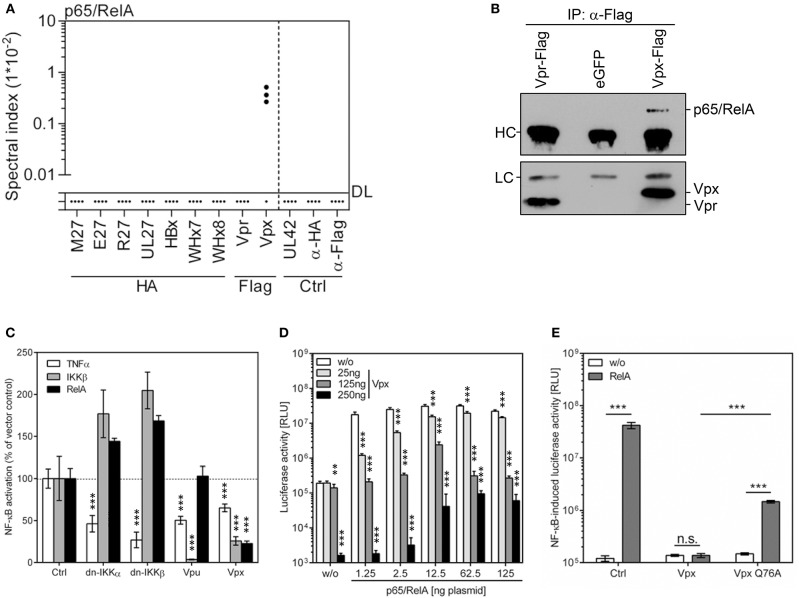
HIV-2 Vpx co-precipitates p65/RelA and inhibits NF-κB signaling. **(A)** Based on the MS data set (see Figure 1B), spectral indexes were calculated for p65/RelA. **(B)** The interaction was validated by IP and subsequent immunoblotting. Vpr served as control. **(C)** Cells were co-transfected with an NF-κB-responsive firefly luciferase reporter construct (100 ng), a *Gaussia* luciferase construct (25 ng) for normalization, and expression vectors for the indicated gene products. NF-κB was activated by TNFα treatment (20 ng/ml) or overexpression of a constitutively active mutant of IKKβ or p65/RelA (both 40 ng). Dominant negative (dn-) IKKα, dn-IKKβ as well as HIV-1 Vpu served as controls (all 100 ng). Luciferase activity was determined 40 h post-transfection. The mean value of 3–9 transfections ± SD is shown. The empty expression plasmid served as control. **(D)** Increasing concentrations of Vpx and p65/RelA expression plasmids were co-transfected with the NF-κB reporter construct, and luciferase activity was determined at 24 h post-transfection. The empty expression plasmid served as control. The experiment was conducted in *n* = 3*3 replicates (three independent transfections, each split and measured in triplicates). A level of significance corresponding to *p* < 0.01 is indicated by “**” whereas *p* < 0.001 is indicated by “***”. **(E)** As above, but wt Vpx and a mutant incapable to interact with DDB1 and CRLs (Q76A) were compared. 2.5 ng p65/RelA and 250 ng plasmid encoding Vpx or Vpx-Q76A were transfected. Student's *t*-Test [two-tailed and heteroscedastic (assuming different variances)] was used to compare the indicated data sets. A level of significance corresponding to *p* < 0.001 is indicated by “***”.

Within the interactome of Vpx, p65 (also called RelA) was the most abundant specifically interacting protein (Figure 6A and Supplementary Table 10). Intrigued by the importance of NF-κB signaling for innate immunity as well as for HIV gene expression, we confirmed this interaction by IP and subsequent immunoblot analysis (Figure 6B). Luciferase-based promoter reporter assays were used to investigate the effects of Vpx on NF-κB-dependent gene expression. NF-κB was activated at different layers: external stimulation by TNFα, stimulation at the level of the IKK complex by the constitutively active IKKβ mutant S177E/S181E, or at the level of p65/RelA by its overexpression. The HIV-1 accessory protein U (Vpu) inhibits NF-κB upstream of p65/RelA (50) and served as positive control. Consistently, Vpu antagonized NF-κB signaling induced by TNFα treatment and expression of constitutively active IKKβ but not by p65/RelA overexpression (Figure 6C). Conversely, Vpx inhibited NF-κB signaling induced by all stimuli (Figure 6C), indicating that Vpx directly acts on p65/RelA. As expected, Vpx inhibits RelA/p65-induced NF-κB activation in a dose-dependent manner (Figure 6D). Since NF-κB signal transduction relies on the β-TrCP/Skp1/Cullin1 complex for IκBα degradation, inhibitors such as MG132 cannot be used to elucidate Ub ligase-dependency of Vpx-mediated NF-κB inhibition. Therefore, the Vpx mutant Q76A, known to lack the ability to interact with VprBP and DDB1 (32), was used to test the relevance of CRLs (Figure 6E). The results suggest that the interaction with DDB1 and CRLs significantly contributes to the NF-κB inhibitory activity of Vpx, although we cannot exclude that the Q76A mutation has pleiotropic effects on Vpx. To test if the interaction of Vpx with DDB1 and RelA/p65 results in the degradation of RelA/p65, we performed co-transfection experiments with Vpx and RelA/p65 expression plasmids. The experiments were conducted in the presence or absence of MG132. MG132 did not change the RelA/p65 abundance, irrespective of the presence of Vpx, and we did not observe decreased RelA/p65 amounts in the presence of Vpx (data not shown). We infected lymphocytes with Vpx-positive and Vpx-negative HIV-2 viruses, and determined RelA/p65 by flow cytometry. Also under these conditions, we did not observe significant changes in the RelA/p65 abundance in the presence of Vpx (data not shown). We hypothesize that Vpx-mediated inhibition of NF-κB signaling cannot be explained sufficiently by proteasomal degradation of RelA/p65. Taken together, our determination of the interactomes of viral accessory proteins uncovered novel interactions with biological relevance.

## Discussion

Viruses frequently dismantle cell intrinsic immunity by evolving accessory proteins that bridge antiviral factors to proteins inducing proteolysis, e.g., DDB1 and CRLs or other components of the Ub proteasome pathway. Since CRLs became amenable for pharmacologic intervention using drugs such as MLN4924 and since CRL activity is essential for replication of several human-pathogenic viruses (21, 72), viral CRL exploitation may be turned into therapeutic approaches. Understanding the exact mechanisms and interactomes of viral accessory proteins might uncover additional and more specific Achilles' heels of viruses.

**Figure 7:**
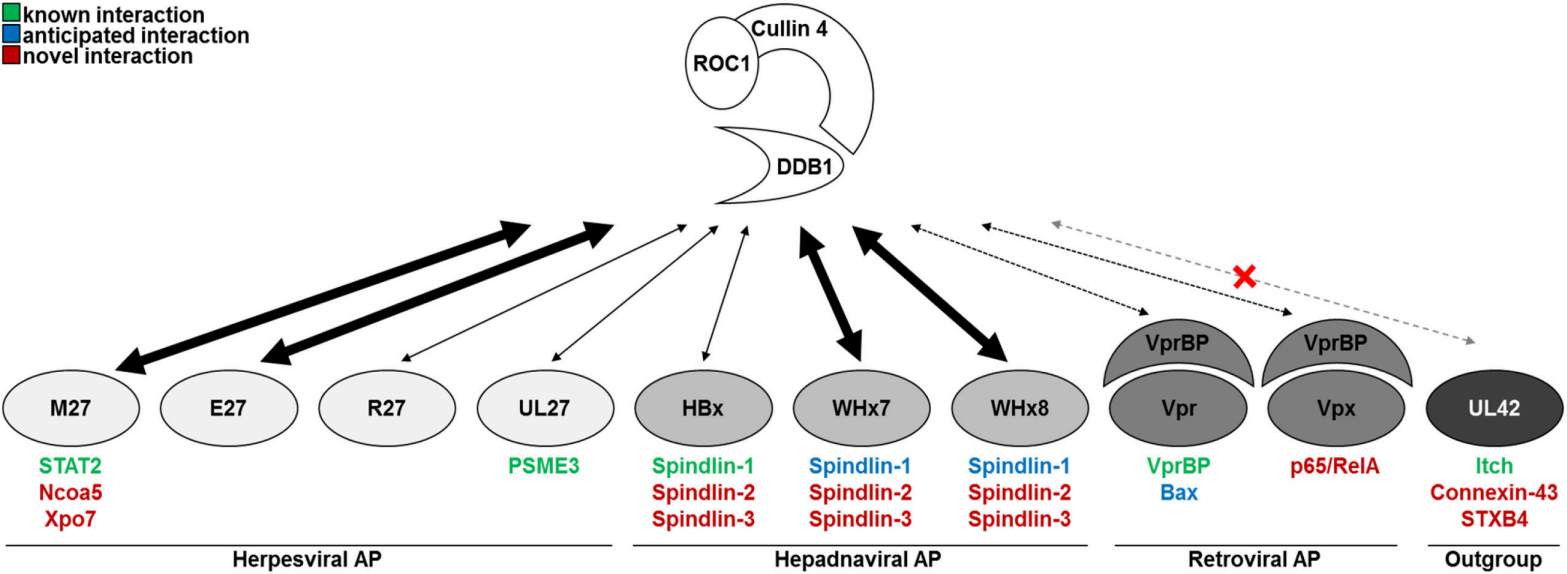
Model and summary of the findings.

Here, we comprehensively compared the interactomes of nine viral accessory proteins known or suspected to interact with DDB1 and/or CRLs and of one outgroup control protein, which did not interact with CRLs but with other Ub ligases of the NEDD4 family (Figure 7). We found that the strength of the interaction between the herpes-, hepadna-, and retroviral accessory proteins and DDB1 and/or CRLs was remarkably different. Despite the fact that the interaction between HBx and DDB1 was confirmed by the co-crystallization of full-length DDB1 with short α-helical peptides corresponding to HBx (39), the interaction turned out to be surprisingly weak in comparison to WHx7, WHx8, pE27, and pM27. This functional difference between HBx and WHx is supported by the existing crystal structure data: the superposition of the DDB1-HBx and DDB1-WHx structures shows that three strong hydrogen bonds are formed between the N-terminal part of WHx and the cognate 4b−4c loop of DDB1, whereas the DDB1 loop is pushed further out in the DDB1-HBx heterodimer only allowing weaker hydrophobic and van der Waals interactions. What might be the reason for this difference? Recent findings documented the existence of stimulatory functions of DDB1 on HBV transcription by HBx-dependent as well as independent mechanisms (73). It is tempting to speculate that the strength of interaction between HBx and DDB1 might represent a virus-specific trade-off between direct effects and HBx-independent functions. Additionally, DDB1 is essential for cell proliferation and survival (74, 75) and some DDB1 functions are impaired by HBx, leading to cell death (76, 77). This suggests that the association between viral accessory proteins and DDB1 needs to balance advantageous and disadvantageous consequences—presumably in a virus-specific manner.

HBx was also shown to interact with Spindlin-1 to alleviate its antiviral effects against HBV (and *Herpesviridae*) (70). We confirmed this interaction and extended it to the WHV-expressed homologs WHx7 and WHx8, indicating evolutionary conservation and functional relevance *in vivo*. Previously described anti- and proviral effects of Spindlin-1 overexpression and siRNA-mediated ablation, respectively, were statistically significant but moderate (below 1^*^log10) (70). Our finding that HBx and WHx also interact with Spindlin-2 and Spindlin-3 suggests redundancy in this recently identified intrinsic defense system.

In addition to hepadnaviral proteins, we also included two lentiviral proteins (i.e., HIV-2 Vpr and Vpx) in our analyses. While HIV-1 Vpr-induced apoptosis occurs in a Bax-dependent manner (78), a direct physical interaction of these two proteins was to our knowledge never substantiated experimentally. The herein shown co-precipitation with HIV-2 Vpr extends the finding from HIV-1 to HIV-2 and fills this gap concerning the physical interaction.

To enable a comparative analysis of the interactomes of the different viral proteins, one cell line was used throughout all experiments. Due to their ubiquitous availability and broad application, we selected HEK293T cells for this purpose. One might argue that viral accessory proteins will usually be expressed in other cell types (e.g., HIV-encoded proteins in CD4+ T lymphocytes or macrophages) and that this experimental approach constitutes a potential confounding factor of our data set. However, HIV can efficiently replicate in HEK293 cells once CD4 expression is enforced (79), suggesting suitability for studies addressing intracellular protein-protein interactions. This is in conjunction with the fact that we confirmed numerous known interactions and were able to validate newly identified interactions under infection conditions (see e.g., Figure 2F).

As a proof of principle for the relevance of the newly identified interactions, we studied the functional consequences of the interaction between HIV-2 Vpx and p65/RelA. Based on the presence of RelA in the Vpx interactome, we tested if Vpx influences NF-κB signal transduction and found that Vpx efficiently inhibited NF-κB activation. To identify the step of the signaling cascade that is targeted by Vpx, we activated NF-κB signaling using different stimuli. Vpx antagonizes NF-κB signaling stimulated by TNFα treatment, expression of constitutively active IKKβ as well as RelA overexpression. Especially the latter result strongly suggests that the interaction between Vpx and RelA is indeed functionally relevant. NF-κB signaling is crucial for the induction and regulation of innate, adaptive, and intrinsic immune responses. Consistently, numerous viruses express proteins, which modulate NF-κB signaling (80). In case of HIV-1 and HIV-2, the situation is complicated by the fact that both viruses contain functional NF-κB binding sites in their LTR promoters which are highly relevant for viral gene expression and replication (81)—although they seem not to be absolutely essential for viral growth (82). It may appear counterintuitive that a virus, which exhibits NF-κB sites in its promoter and which exploits NF-κB signaling for its own transcription, should express an antagonist targeting RelA. However, NF-κB inhibitory effects are clearly documented for the accessory proteins Vpu and Vpr of HIV-1 and related primate lentiviruses (50, 83–87). These NF-κB inhibitory activities may confer a selection advantage as they prevent the NF-κB-driven expression of anti-viral cytokines [e.g., IFNs and Rantes (83)] and restriction factors during late stages of the viral replication cycle when efficient expression of viral genes has already been initiated.

Taken together, our comparative study on the interactome of viral accessory proteins known or suspected to interact with DDB1 and CRLs uncovered several new aspects of the biology of these important proteins. Furthermore, our data constitute a rich resource for future studies addressing the molecular functions and mechanisms of these viral proteins.

## Data Availability Statement

The proteomic datasets generated for this study can be found in the ProteomeXchange Consortium (http://proteomecentral.proteomexchange.org) (52) via the PRIDE partner repository (53) with the data identifier PXD007634 and DOI 10.6019/PXD007634.

## Author Contributions

CL, MR, ME, JR-A, SeH, StH, and VTKL-T did experiments. DM and BS performed MS quantifications. DH and DS analyzed the effect of HIV-2 Vpx on NF-κB activation. CL, VTKL-T, and MT wrote the paper. VTKL-T and MT supervised the project.

### Conflict of Interest Statement

The authors declare that the research was conducted in the absence of any commercial or financial relationships that could be construed as a potential conflict of interest.
