# Supplementary material for: A Mass Spectrometry-Based Profiling of Interactomes of Viral DDB1- and Cullin Ubiquitin Ligase-Binding Proteins Reveals NF-κB Inhibitory Activity of the HIV-2-Encoded Vpx
Source: Front Immunol. 2018 Dec 19;9:2978. doi: 10.3389/fimmu.2018.02978 (PMC6305766; doi:10.3389/fimmu.2018.02978)
Supplement: Supplementary file 1 [file Data_Sheet_1.PDF]

A Most abundant interaction partners of pUL42-HA

No.	UniProt	Protein	Coverage				Unique peptides				Replicates (x/4)	Spectral Index
1	P38646	GRP75	37.56	34.90	36.82	35.79	21	16	17	17	4	0.07650
2	Q96J02	ITCH	29.46	10.52	16.83	7.97	15	7	9	4	4	0.03357
3	P08107	HS71A	33.70	25.12	29.64	16.22	12	11	11	6	4	0.03012
4	P11021	GRP78	38.23	31.19	32.72	23.70	21	15	15	12	4	0.02756
5	P11142	HSP7C	39.78	34.21	30.34	42.11	16	11	10	4	4	0.01262
6	O00308	WWP2	19.20	9.89	14.83	2.64	10	6	7	2	4	0.01188
7	Q96PU5	NED4L	17.23	3.83	8.21	6.05	11	2	5	4	4	0.01108
8	P46934	NEDD4	10.77	1.65	5.53	3.74	8	1	4	2	4	0.00852
9	Q13885	TBB2A	27.64	0.00	23.15	0.00	1	0	1	0	2	0.00817
10	Q6ZWJ1	STXBP4	17.90	9.98	9.79	2.64	8	4	4	1	4	0.00794
11	Q9H0M0	WWP1	12.58	0.00	5.53	2.93	5	0	1	1	3	0.00707
12	J3QSA3	UBB	48.84	67.44	67.44	0.00	2	2	2	0	3	0.00454
13	P04350	TBB4A	26.58	0.00	0.00	8.33	1	0	0	1	2	0.00398
14	P08670	VIME	30.26	12.53	21.24	4.64	12	4	8	2	4	0.00354
15	O95817	BAG3	10.43	4.70	4.70	0.00	4	2	2	0	3	0.00287
16	P17302	Connexin-43	23.82	7.59	3.93	0.00	5	2	1	0	3	0.00285
17	K7EKL3	GRN	6.96	5.93	5.93	11.82	2	2	2	1	4	0.00276
18	O00560	SDCB1	14.09	14.09	14.09	0.00	2	2	2	0	3	0.00233
19		UL42-HA	17.16	17.16	17.16	0.00	1	1	1	0	3	0.00207
20	Q9H0U4	RAB1B	22.89	9.47	9.47	0.00	2	1	1	0	3	0.00202

B Most abundant exclusive interaction partners of pUL42-HA

No.	UniProt	Protein	Coverage				Unique peptides				Replicates (x/4)	Spectral Index
1	P46934	NEDD4	10.77	1.65	5.53	3.74	8	1	4	2	4	0.00852
2	Q6ZWJ1	STXBP4	17.90	9.98	9.79	2.64	8	4	4	1	4	0.00794
3	Q9H0M0	WWP1	12.58	0.00	5.53	2.93	5	0	1	1	3	0.00707
4	O95817	BAG3	10.43	4.70	4.70	0.00	4	2	2	0	3	0.00287
5	P17302	Connexin-43	23.82	7.59	3.93	0.00	5	2	1	0	3	0.00285
6	O00560	SDCB1	14.09	14.09	14.09	0.00	2	2	2	0	3	0.00233
7	Q8IZ07	AN13A	9.49	8.47	4.24	0.00	4	4	2	0	3	0.00197
8	P46937	YAP1	25.40	27.91	3.57	0.00	6	3	1	0	3	0.00146
9	Q15738	NSDHL	12.60	4.29	4.29	0.00	2	1	1	0	3	0.00070
10	Q9Y3Y2	CHTOP	5.24	5.24	5.24	0.00	1	1	1	0	3	0.00058
11	Q96KB7	CIZ1	6.52	0.00	2.90	0.00	2	0	1	0	2	0.00031
12	H0YGG7	H0YGG7	0.00	24.19	24.19	0.00	0	1	1	0	2	0.00031
13	P01593	KVD33	0.00	16.67	16.67	0.00	0	1	1	0	2	0.00031
14	K7ENK9	VAMP2	0.00	25.00	25.00	0.00	0	1	1	0	2	0.00031
15	O75131	CPNE3	6.67	1.68	0.00	0.00	1	1	0	0	2	0.00027

- Family member of Nedd4-like Ub ligases harboring a WW domain which binds PPxY motifs
- Protein with WW domain
- Known NEDD4 substrate
- Protein with PPxY motif

Suppl.-Fig. S1: Interaction partners of HCMV pUL42

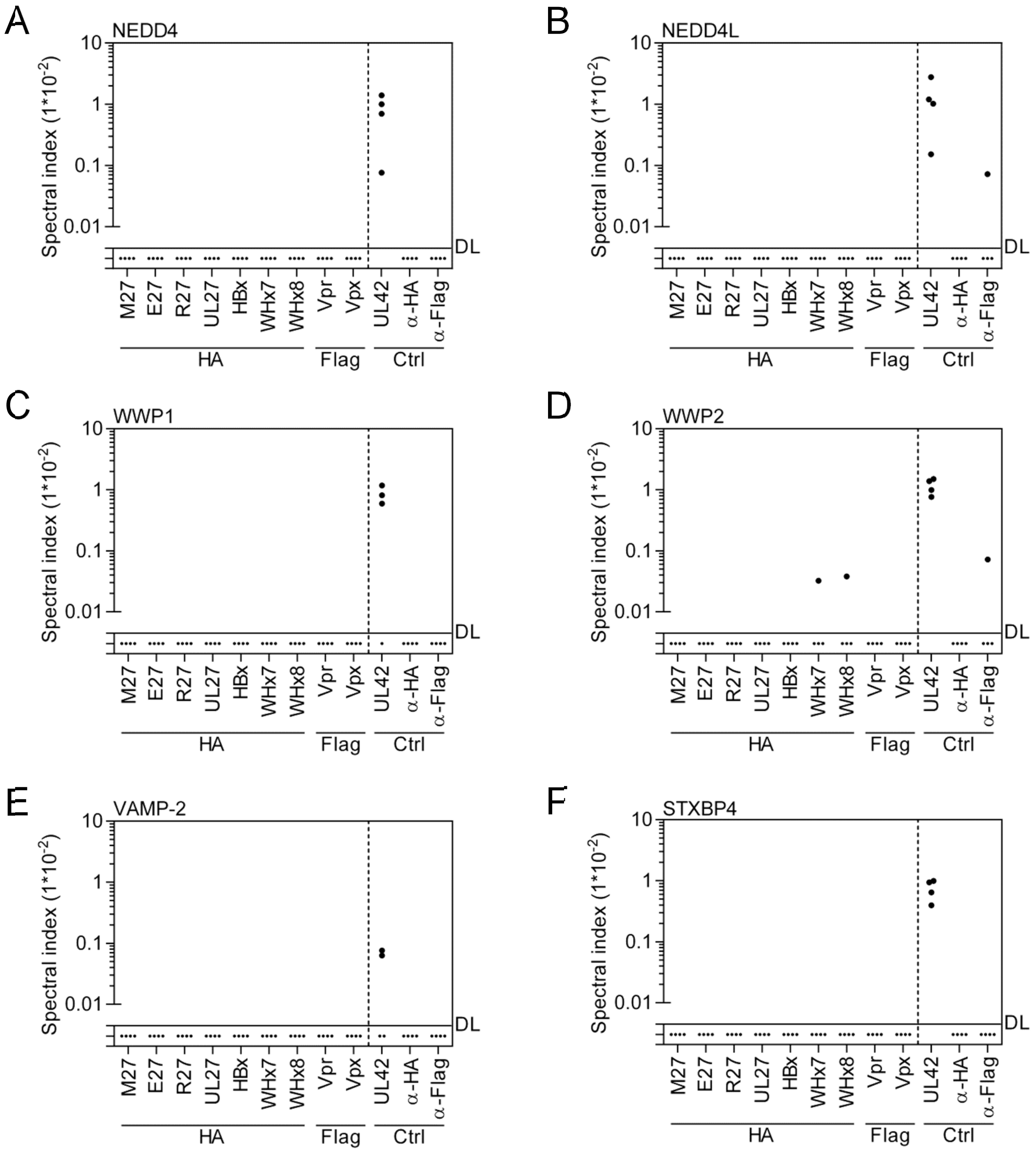

Suppl.-Fig. S2: HCMV pUL42 interacts with WW domain-containing proteins like NEDD4 Ub ligase family members

A Most abundant interaction partners of pM27-HA

No.	UniProt	Protein	Coverage				Unique peptides				Replicates (x/4)	Spectral Index	Reference (PMID)
1		M27-HA	42.55	37.77	37.05	35.17	26	20	19	20	4	0.11413	
2	Q16531	DDB1	32.72	11.49	18.33	20.88	33	9	16	21	4	0.04931	21698215
3	Q13885	TBB2A	35.73	28.09	23.60	28.54	2	1	2	1	4	0.02480	
4	Q9HCD5	NCOA5	16.58	14.51	11.92	23.83	5	4	4	6	4	0.00961	
5	K4DI93	CUL4B	24.11	12.44	14.78	15.33	13	7	7	8	4	0.00845	21698215
6	Q5T9A4	ATD3B	28.55	13.58	17.13	14.81	9	4	4	3	4	0.00785	
7	P07437	TBB5	46.62	29.34	27.46	29.81	4	3	3	3	4	0.00785	
8	Q9NVI7	ATD3A	24.65	13.88	18.93	12.41	6	3	4	2	4	0.00706	
9	P52597	HNRPF	24.82	22.17	21.45	15.90	4	5	4	3	4	0.00640	
10	P68371	TBB4B	46.52	28.09	26.29	28.54	1	1	3	3	4	0.00594	
11	Q13619	CUL4A	20.42	6.06	10.41	8.96	8	3	4	3	4	0.00544	21698215
12	Q9UIA9	XPO7	21.16	4.78	8.28	11.96	17	4	7	9	4	0.00485	
13	P04350	TBB4A	44.82	17.57	0.00	0.00	1	1	0	0	2	0.00460	
14	O14654	IRS4	19.25	8.83	7.88	6.36	15	6	6	5	4	0.00401	
15	Q9BUF5	TBB6	30.94	23.99	15.25	20.18	5	5	2	2	4	0.00398	
16	P14373	TRI27	24.17	16.18	18.52	12.48	8	5	6	4	4	0.00396	
17	P78371	TCPB	35.70	20.67	28.13	15.38	12	6	8	4	4	0.00377	
18	E9PCY7	HNRH1	23.54	0.00	21.45	10.49	3	0	2	2	3	0.00324	
19	P52272	HNRPM	16.71	18.36	15.21	15.21	9	9	6	6	4	0.00319	
20	A8MTG8	ARMC8	19.00	10.26	13.27	13.33	10	6	7	1	4	0.00305	

B Most abundant exclusive interaction partners of pM27-HA

No.	UniProt	Protein	Coverage				Unique peptides				Replicates (x/4)	Spectral Index	Reference (PMID)
1	Q9HCD5	NCOA5	16.58	14.51	11.92	23.83	5	4	4	6	4	0.00961	
2	Q9UIA9	XPO7	21.16	4.78	8.28	11.96	17	4	7	9	4	0.00485	
3	Q5BJF6	ODFP2	6.88	2.90	4.34	3.26	5	2	3	2	4	0.00114	
4	Q96CN9	GCC1	4.26	1.68	2.71	1.03	3	1	2	1	4	0.00067	
5	F8VUX5	KANSL2	8.53	14.29	14.29	14.29	2	1	1	1	4	0.00048	
6	P52630	STAT2	10.93	2.17	2.17	0.00	6	1	1	0	3	0.00048	21698215
7	P85037	FOXK1	6.14	3.68	0.00	0.00	2	1	0	0	2	0.00029	
8	B4DH70	FBXW11	0.00	2.16	2.16	0.00	0	1	1	0	2	0.00019	
9	H7BZT4	HT7BZT4	0.00	12.63	12.63	0.00	0	1	1	0	2	0.00019	
10	Q5TDG9	DJC16	1.34	1.68	0.00	0.00	1	1	0	0	2	0.00019	
11	Q01167	FOXK2	2.58	0.00	2.58	0.00	1	0	1	0	2	0.00019	
12	Q9UKL0	RCOR1	3.32	2.90	0.00	0.00	1	1	0	0	2	0.00019	
13	Q8NF99	ZN397	1.87	0.00	7.19	0.00	1	0	1	0	2	0.00019	

Suppl.-Fig. S3: Interaction partners of MCMV pM27

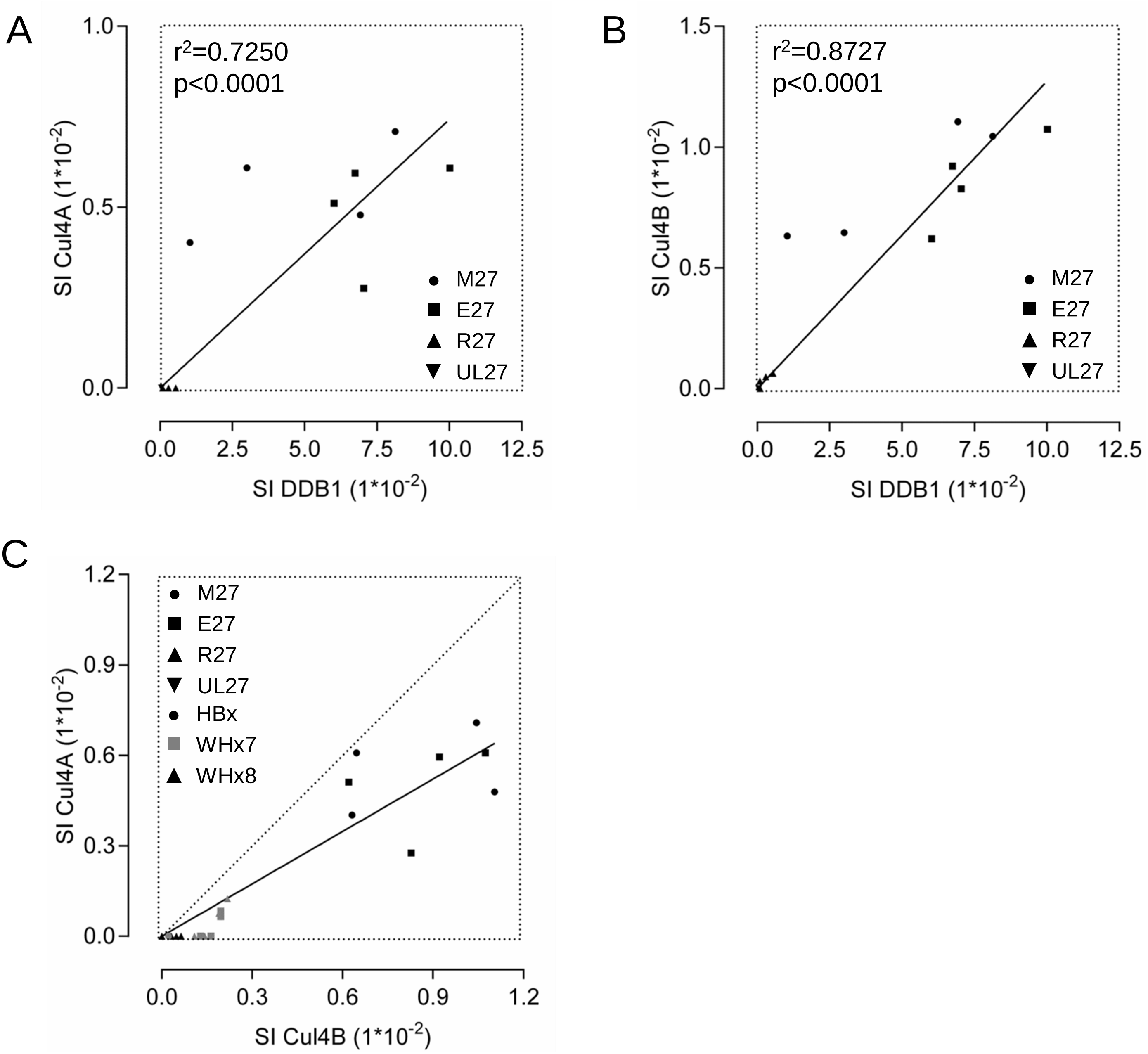

Suppl.-Fig. S4: Correlation between the co-precipitation of DDB1, Cul4A, and Cul4B

A Most abundant interaction partners of pUL27-HA

No.	UniProt	Protein	Coverage				Unique peptides				Replicates (x/4)	Spectral Index
1	Q13885	TBB2A	38.88	32.58	23.60	26.29	1	1	1	1	4	0.02790
2		UL27-HA	19.12	18.48	16.53	16.37	9	8	7	6	4	0.02722
3	P04350	TBB4A	32.43	25.00	0.00	24.55	1	1	0	1	3	0.01888
4	P07437	TBB5	44.37	32.63	27.46	27.46	4	2	3	3	4	0.01378
5	P68371	TBB4B	38.20	34.61	26.29	26.29	1	1	3	1	4	0.01214
6	Q14257	RCN2	41.64	41.32	37.22	37.22	10	9	7	7	4	0.01180
7	P61289	PSME3	46.46	37.01	37.01	37.01	10	7	7	7	4	0.01156
8	Q9BVA1	TBB2B	0.00	32.58	23.60	26.29	0	1	1	1	3	0.01095
9	Q9BUF5	TBB6	26.91	23.54	15.25	21.30	5	4	2	4	4	0.00690
10	Q9NVI7	ATD3A	28.39	17.35	14.04	13.64	7	2	1	3	4	0.00640
11	Q13263	TIF1B	29.22	12.93	21.56	15.21	13	5	10	8	4	0.00472
12	P52272	HNRPM	27.68	17.26	19.86	19.86	7	6	6	10	4	0.00472
13	A8MTG8	ARMC8	15.54	12.52	14.93	13.12	8	7	8	7	4	0.00324
14	O14654	IRS4	14.88	8.35	8.83	7.40	11	6	6	6	4	0.00316
15	O43852	CALU	37.14	12.38	10.79	19.05	8	3	3	5	4	0.00306
16	Q9BQE3	TBA1C	39.20	37.42	34.74	0.00	1	1	1	0	3	0.00284
17	P68363	TBA1B	39.47	37.69	35.03	29.49	1	1	1	8	4	0.00275
18	Q7L804	RFIP2	23.12	16.60	17.19	16.99	9	6	6	6	4	0.00272
19	B4DQH9	RBP10	17.68	9.09	9.93	11.78	5	4	3	4	4	0.00258
20	E9PCY7	HNRH1	23.54	14.22	14.22	14.22	3	1	3	1	4	0.00253
167	Q16531	DDB1	5.68	4.17	1.83	1.83	3	1	2	2	4	0.00051

B Most abundant exclusive interaction partners of pUL27-HA

No.	UniProt	Protein	Coverage				Unique peptides				Replicates (x/4)	Spectral Index
1	K7ERP6	KAPCA	21.12	11.55	11.55	6.77	3	2	2	1	4	0.00106
2	Q9NXF1	TEX10	9.26	1.40	3.44	3.44	6	1	2	2	4	0.00105
3	H3BQQ6	F192A	26.04	26.04	26.04	26.04	2	2	2	2	4	0.00082
4	Q8IVF7	FMNL3	5.84	0.00	2.24	2.24	5	0	2	2	3	0.00082
5	Q9BVS4	RIOK2	5.07	5.07	5.07	5.07	2	2	2	2	4	0.00082
6	F5GYL3	DDX24	10.54	0.00	6.90	1.97	6	0	3	1	3	0.00078
7	Q8TAA9	VANG1	8.40	0.00	3.63	5.92	3	0	1	2	3	0.00059
8	O15355	PPM1G	5.31	3.11	0.00	3.11	2	1	0	1	3	0.00049
9	Q99614	TTC1	11.30	6.13	0.00	6.85	2	1	0	1	3	0.00049
10	P10074	TZAP	5.23	8.90	0.00	8.90	2	1	0	1	3	0.00049
11	F8VYK6	TRBP2	13.64	13.64	13.64	22.73	1	1	1	2	4	0.00044
12	Q0D2I5	IFFO1	0.00	1.97	2.33	1.97	0	1	1	1	3	0.00041
13	Q13164	MK07	5.88	0.00	8.33	8.33	3	0	1	1	3	0.00041
14	Q6UXN9	WDR82	6.39	6.39	0.00	6.39	1	1	0	1	3	0.00036
15	Q8NB46	ANR52	1.49	0.00	0.93	0.93	1	0	1	1	3	0.00032
16	F8VRQ9	CACO1	9.38	0.00	9.38	9.38	1	0	1	1	3	0.00032
17	K7ELH7	GID4	11.71	11.71	11.71	0.00	1	1	1	0	3	0.00032
18	U3KQC1	WDR18	10.66	5.29	0.00	0.00	3	1	0	0	2	0.00026
19	H3BS42	ZN768	0.00	4.72	0.00	2.75	0	2	0	1	2	0.00023
20	Q5T8C6	CDC16	0.00	0.00	3.16	3.16	0	0	1	1	2	0.00018
21	Q9NYW8	RBAK	3.78	0.00	1.54	0.00	2	0	1	0	2	0.00018
22	Q15906	VPS72	0.00	3.30	3.30	0.00	0	1	1	0	2	0.00018
23	P17014	ZNF12	0.00	0.00	1.72	1.72	0	0	1	1	2	0.00018
24	M0R0T4	ZN714	0.00	0.00	5.39	5.39	0	0	1	1	2	0.00018
25	C9IZS8	ZNF92	0.00	3.92	1.96	0.00	0	2	1	0	2	0.00018
26	Q5JYG3	GPN2	12.21	0.00	12.21	0.00	1	0	1	0	2	0.00014
27	D6RE80	MAEA	7.65	0.00	0.00	4.92	1	0	0	1	2	0.00014
28	F2Z2K0	NSF1C	5.84	5.84	0.00	0.00	1	1	0	0	2	0.00014
29	H0YLG5	RMD3	3.83	0.00	0.00	13.94	1	0	0	2	2	0.00014
30	H0YDA9	RSBN1	3.81	0.00	3.81	0.00	1	0	1	0	2	0.00014

Suppl.-Fig. S5: Interaction partners of HCMV pUL27

A Most abundant interaction partners of Vpr-Flag

No.	UniProt	Protein	Coverage				Unique peptides				Replicates (x/4)	Spectral Index
1	P04264	K2C1	32.45	20.81	6.99	31.83	12	9	4	15	4	0.03741
2	P13645	K1C10	40.58	35.62	2.05	35.10	18	14	1	15	4	0.03322
3	P38646	GRP75	42.12	35.64	17.82	42.27	24	19	8	23	4	0.01097
4	P01617	KVD28	11.50	11.50	11.50	11.50	1	1	1	1	4	0.01062
5	Q13885	TBB2A	29.89	26.29	0.00	0.00	1	1	0	0	2	0.00698
6	Q9BVA1	TBB2B	29.89	26.29	0.00	0.00	1	1	0	0	2	0.00698
7	P04350	TBB4A	22.97	20.72	0.00	0.00	1	1	0	0	2	0.00620
8	P35908	K22E	41.00	27.86	0.00	33.33	13	10	0	11	3	0.00347
9	P02533	K1C14	37.50	30.72	0.00	22.88	9	6	0	4	3	0.00346
10	P08779	K1C16	39.11	19.45	0.00	19.45	9	3	0	3	3	0.00297
11	Q04695	K1C17	33.33	14.81	0.00	9.95	9	1	0	2	3	0.00163
12	P68104	EF1A1	12.77	7.58	0.00	2.38	4	2	0	1	3	0.00139
13	O00571	DDX3X	9.82	8.16	0.00	0.00	4	3	0	0	2	0.00129
14	P32455	GBP1	2.03	2.03	0.00	0.00	1	1	0	0	2	0.00129
15	Q9BW61	DDA1	27.45	27.45	0.00	15.69	2	2	0	1	3	0.00129
16	Q9Y4B6	VPRBP	3.65	2.39	0.00	0.00	5	3	0	0	2	0.00116
17	Q8N1N4	K2C78	11.15	9.04	0.00	1.73	4	4	0	1	3	0.00099
18	P01834	IGKC	49.06	32.08	0.00	18.87	3	2	0	1	3	0.00099
19	Q16531	DDB1	9.21	5.61	0.00	11.09	6	4	0	3	3	0.00096
20	J3QSA3	UBB	37.21	37.21	0.00	37.21	1	1	0	1	3	0.00089

B Most abundant exclusive interaction partners of Vpr-Flag

No.	UniProt	Protein	Coverage				Unique peptides				Replicates (x/4)	Spectral Index
1	Q07812	BAX	12.50	6.77	0.00	6.77	2	1	0	1	3	0.00078
2	P47929	LEG7	11.76	0.00	0.00	8.09	1	0	0	1	2	0.00026

Suppl.-Fig. S6: Interaction partners of HIV-2 Vpr
