# Supplementary material for: A Mass Spectrometry-Based Profiling of Interactomes of Viral DDB1- and Cullin Ubiquitin Ligase-Binding Proteins Reveals NF-κB Inhibitory Activity of the HIV-2-Encoded Vpx
Source: Front Immunol. 2018 Dec 19;9:2978. doi: 10.3389/fimmu.2018.02978 (PMC6305766; doi:10.3389/fimmu.2018.02978)
Supplement: Supplementary file 2 [file Data_Sheet_2.PDF]

A Most abundant interaction partners of pE27-HA

No.	UniProt	Protein	Coverage				Unique peptides				Replicates (x/4)	Spectral Index
1		E27-HA	44.06	39.85	27.07	40.00	27	24	15	22	4	0.11197
2	Q16531	DDB1	33.60	24.91	21.40	23.95	34	25	19	24	4	0.06860
3	Q13885	TBB2A	43.15	33.48	0.00	24.49	1	1	0	1	3	0.02264
4	P07437	TBB5	43.24	37.09	29.81	28.40	4	3	3	3	4	0.01388
5	P68371	TBB4B	43.15	35.51	28.54	27.19	1	1	3	2	4	0.01086
6	Q9BVA1	TBB2B	43.15	33.48	28.54	24.49	1	1	2	1	4	0.01047
7	K4DI93	CUL4B	30.00	16.56	13.56	18.00	16	9	7	9	4	0.00875
8	P04350	TBB4A	36.49	28.83	0.00	0.00	2	1	0	0	2	0.00654
9	P78371	TCPB	41.87	35.14	16.79	23.74	15	12	5	8	4	0.00598
10	Q9NVI7	ATD3A	23.34	14.69	9.62	9.62	6	4	1	1	4	0.00578
11	Q13619	CUL4A	19.89	12.38	6.06	7.25	9	7	2	2	4	0.00553
12	P10809	CH60	42.06	28.62	23.73	21.47	14	11	9	8	4	0.00404
13	P50990	TCPQ	21.93	23.72	16.45	17.39	10	11	7	8	4	0.00385
14	O14654	IRS4	16.47	10.82	5.33	4.53	12	8	4	3	4	0.00356
15	P17987	TCPA	36.33	22.66	15.47	13.67	14	9	5	5	4	0.00355
16	P68363	TBA1B	0.00	37.69	31.49	25.28	0	1	1	7	3	0.00319
17	E9PCY7	HNRH1	18.65	23.54	18.65	10.49	2	3	4	2	4	0.00304
18	P40227	TCPZ	19.21	18.08	11.60	11.60	6	6	3	3	4	0.00295
19	Q99832	TCPH	21.55	24.49	12.71	9.76	9	9	4	3	4	0.00286
20	B4DUR8	TCPG	22.20	6.60	15.60	11.00	9	4	6	5	4	0.00286

B Most abundant exclusive interaction partners of pE27-HA

No.	UniProt	Protein	Coverage				Unique peptides				Replicates (x/4)	Spectral Index
1	B4DZ20	ELMO2	25.07	11.00	7.71	10.18	11	5	2	3	4	0.00271
2	Q9BVL4	SELO	3.74	3.74	0.00	1.79	2	2	0	1	3	0.00048
3	Q14185	DOCK1	1.50	0.75	0.00	0.00	2	1	0	0	2	0.00018
4	Q92556	ELMO1	3.99	1.65	0.00	0.00	2	1	0	0	2	0.00018
5	Q9NQX0	PRDM6	2.35	2.35	0.00	0.00	1	1	0	0	2	0.00015

Suppl. Table 1: Interaction partners of RCMV pE27

A Most abundant interaction partners of pR27-HA

No.	UniProt	Protein	Coverage				Unique peptides				Replicates (x/4)	Spectral Index
1	Q13885	TBB2A	48.54	33.48	28.54	25.84	2	1	1	1	4	0.03276
2		R27-HA	30.00	28.09	26.76	22.94	17	14	13	11	4	0.02435
3	P07437	TBB5	55.86	34.98	37.32	37.32	4	3	3	3	4	0.01997
4	P68371	TBB4B	55.73	33.48	35.73	35.73	1	1	3	3	4	0.01753
5	Q9BVA1	TBB2B	0.00	33.48	28.54	25.84	0	1	1	1	3	0.01510
6	Q9NVI7	ATD3A	33.57	32.17	15.62	8.68	7	6	1	2	4	0.01079
7	P04350	TBB4A	50.00	26.80	0.00	0.00	1	1	0	0	2	0.00893
8	P78371	TCPB	35.14	32.90	29.91	23.96	12	11	10	8	4	0.00761
9	Q9BQE3	TBA1C	39.87	37.42	31.18	28.06	1	1	1	2	4	0.00731
10	P52272	HNRPM	27.68	27.68	18.36	15.21	7	7	6	6	4	0.00700
11	P68363	TBA1B	41.91	39.47	31.49	0.00	2	2	1	0	3	0.00666
12	Q9BUF5	TBB6	23.77	23.54	20.18	17.49	4	3	2	2	4	0.00616
13	B4DEM7	TCPQ	26.65	24.76	20.79	20.42	12	11	9	9	4	0.00528
14	P17987	TCPA	24.28	34.89	22.12	22.30	9	12	8	8	4	0.00513
15	Q13263	TIF1B	28.38	17.72	22.83	23.70	14	9	6	6	4	0.00491
16	O14654	IRS4	14.96	12.65	6.36	9.71	11	9	5	7	4	0.00469
17	E9PCY7	HNRH1	23.54	25.87	19.11	10.49	3	3	2	2	4	0.00368
18	B4DUR8	TCPG	26.00	18.20	20.00	9.20	11	8	8	4	4	0.00357
19	Q99832	TCPH	21.36	20.44	18.23	13.08	8	7	6	4	4	0.00336
20	Q14257	RCN2	35.96	33.75	33.75	23.66	7	6	6	4	4	0.00336
44	Q16531	DDB1	11.32	5.96	8.65	10.18	9	3	3	7	4	0.00165

B Most abundant exclusive interaction partners of pR27-HA

No.	UniProt	Protein	Coverage				Unique peptides				Replicates (x/4)	Spectral Index
1	Q12955	ANK3	0.87	6.23	6.23	0.34	3	1	1	1	4	0.00033
2	P52756	RBM5	1.72	10.00	1.60	0.00	1	1	1	0	3	0.00028
3	B4DZV1	TTC31	5.50	5.50	5.50	0.00	1	1	1	0	3	0.00028
4	Q01804	OTUD4	0.00	0.00	1.44	1.44	0	0	1	1	2	0.00016
5	Q5TGY3	AHDC1	0.00	1.19	0.00	2.18	0	1	0	2	2	0.00016
6	Q15699	ALX1	7.67	3.99	0.00	0.00	2	1	0	0	2	0.00016
7	M0R2J0	CYH2	11.21	6.54	0.00	0.00	2	1	0	0	2	0.00016
8	Q9UBS3	DNJB9	10.76	6.28	0.00	0.00	2	1	0	0	2	0.00016
9	Q96BN2	TADA1	8.06	3.28	0.00	0.00	2	1	0	0	2	0.00016
10	Q5T8Z2	BMI1	9.24	0.00	0.00	9.24	1	0	0	1	2	0.00012
11	Q5T4Y8	CNKR3	7.82	0.00	7.82	0.00	1	0	1	0	2	0.00012
12	O43248	HXC11	8.88	8.88	0.00	0.00	1	1	0	0	2	0.00012
13	Q96JN0	LCOR	3.93	0.00	3.93	0.00	1	0	1	0	2	0.00012
14	H0YM21	MO4L1	7.69	18.92	0.00	0.00	1	1	0	0	2	0.00012
15	F8W9Y9	MTA1	4.71	4.71	0.00	0.00	1	1	0	0	2	0.00012
16	Q9Y2Z2	MTO1	1.67	9.57	0.00	0.00	1	1	0	0	2	0.00012
17	I3L0I1	SOGA1	1.19	1.42	0.00	0.00	1	1	0	0	2	0.00012
18	Q8NCJ5	SPRY3	2.49	2.49	0.00	0.00	1	1	0	0	2	0.00012
19	E7ERI3	TARS	1.99	1.99	0.00	0.00	1	1	0	0	2	0.00012
20	O94874	UFL1	1.51	1.51	0.00	0.00	1	1	0	0	2	0.00012
21	O43298	ZBT43	6.63	2.78	0.00	0.00	1	1	0	0	2	0.00012
22	Q7L945	ZN627	4.12	4.12	0.00	0.00	1	1	0	0	2	0.00012

Suppl. Table 2: Interaction partners of RCMV pR27

A Most abundant interaction partners of HBx-HA

No.	UniProt	Protein	Coverage				Unique peptides				Replicates (x/4)	Spectral Index
1	Q13885	TBB2A	45.84	30.34	27.19	30.34	1	1	1	1	4	0.04067
2	P07437	TBB5	47.97	38.51	35.92	39.20	4	4	3	3	4	0.02906
3	P68371	TBB4B	41.80	32.36	34.38	37.53	1	1	1	3	4	0.02717
4	P04350	TBB4A	40.09	26.58	31.76	0.00	2	1	1	0	3	0.02687
5	Q9BVA1	TBB2B	45.84	30.34	27.19	30.34	1	1	1	1	4	0.02463
6	Q9BQE3	TBA1C	41.65	43.43	37.42	35.63	2	1	1	2	4	0.02240
7	Q9BUF5	TBB6	38.79	21.30	23.54	17.94	6	4	5	2	4	0.01240
8	P10809	CH60	43.98	28.62	27.05	28.27	15	11	10	10	4	0.01047
9		HBx-HA	25.15	24.54	24.54	24.54	4	3	3	3	4	0.00840
10	Q71U36	TBA1A	47.23	0.00	0.00	35.92	3	0	0	2	2	0.00682
11	Q14257	RCN2	32.81	33.75	23.66	33.75	8	6	4	6	4	0.00328
12	O14654	IRS4	13.13	10.82	8.11	5.89	9	8	6	4	4	0.00293
13	P52272	HNRPM	23.70	18.36	20.89	13.70	7	9	6	7	4	0.00287
14	P08670	VIME	42.92	22.96	44.64	21.24	16	9	18	8	4	0.00267
15	P68363	TBA1B	0.00	43.68	39.47	0.00	0	1	2	0	2	0.00258
16	E9PCY7	HNRH1	23.54	14.22	14.22	12.27	3	1	3	1	4	0.00251
17	P08107	HSP71	39.78	35.10	31.51	32.92	15	13	13	12	4	0.00249
18	Q96EY1	DNJA3	11.88	11.67	13.54	9.17	4	4	4	3	4	0.00219
19	G3V576	HNRPC	44.16	8.23	41.03	0.00	1	2	4	0	3	0.00194
20	B1ANR0	PABP4	6.50	6.50	6.50	6.50	1	1	1	1	4	0.00194
80	Q16531	DDB1	4.67	0.00	3.86	3.51	3	0	3	3	3	0.00079

B Most abundant exclusive interaction partners of HBx-HA

No.	UniProt	Protein	Coverage				Unique peptides				Replicates (x/4)	Spectral Index
1	Q3SY69	AL1L2	11.16	5.53	2.82	8.02	8	4	2	6	4	0.00161
2	B4DWB0	NUBPL	12.31	16.14	11.98	11.98	1	2	1	1	4	0.00035
3	Q8NG31	KNL1	0.00	0.68	0.68	0.68	0	1	1	1	3	0.00033
4	Q8IYQ7	THNS1	2.02	2.02	0.00	2.02	1	1	0	1	3	0.00029
5	Q5JTZ9	SYAM	1.32	0.00	1.32	1.32	1	0	1	1	3	0.00027
6	O75616	ERAL1	6.18	4.35	0.00	0.00	2	1	0	0	2	0.00018
7	C9J7X6	LCHN	0.00	3.70	0.00	3.70	0	1	0	1	2	0.00018
8	M0QZS6	SAE1	11.70	0.00	34.00	0.00	2	0	1	0	2	0.00015
9	H0YDD4	DLAT	3.55	3.55	0.00	0.00	1	1	0	0	2	0.00011
10	Q8TCT9	HM13	3.18	3.18	0.00	0.00	1	1	0	0	2	0.00011
11	F8WDV0	IPO11	1.04	0.00	0.00	1.62	1	0	0	1	2	0.00011
12	S4R446	MEX3D	3.88	3.88	0.00	0.00	1	1	0	0	2	0.00011
13	Q9NX31	OSER1	6.16	0.00	0.00	6.16	1	0	0	1	2	0.00011

Suppl. Table 3: Interaction partners of HBV HBx

A Most abundant interaction partners of WHx7-HA

No.	UniProt	Protein	Coverage				Unique peptides				Replicates (x/4)	Spectral Index
1	Q13885	TBB2A	46.52	34.16	29.66	29.44	1	1	1	1	4	0.04420
2	P04350	TBB4A	35.81	30.41	34.23	33.11	2	2	1	1	4	0.03737
3	P07437	TBB5	48.65	36.26	38.50	38.26	4	3	3	3	4	0.03425
4	Q9BVA1	TBB2B	46.52	34.16	29.66	29.44	1	1	1	1	4	0.02821
5	P68371	TBB4B	42.47	36.18	36.85	36.63	1	1	1	1	4	0.02805
6	P68363	TBA1B	0.00	37.69	42.13	32.82	0	1	1	1	3	0.02266
7	Q9BQE3	TBA1C	44.77	37.42	41.87	32.52	1	1	1	1	4	0.02226
8	Q16531	DDB1	17.02	13.60	15.53	18.42	16	11	13	15	4	0.01543
9	Q9BUF5	TBB6	26.91	23.54	23.77	20.18	5	5	4	2	4	0.01276
10	P08670	VIME	44.64	43.56	19.53	28.97	18	17	7	10	4	0.01136
11		WHx7-HA	23.33	24.00	16.00	16.67	3	3	2	2	4	0.00363
12	O14654	IRS4	9.39	10.26	10.66	7.88	7	7	8	6	4	0.00299
13	O60814	H2B1K	30.16	19.84	11.90	11.90	1	1	1	1	4	0.00286
14	G3V2H6	HNRPC	33.97	33.97	22.22	34.85	9	9	1	2	4	0.00271
15	P62805	H4	33.98	33.01	21.36	21.36	4	3	2	2	4	0.00259
16	P31689	DNJA1	27.96	23.43	15.11	14.58	7	6	4	2	4	0.00251
17	Q96KK5	H2A1H	21.88	21.88	21.88	21.88	1	1	1	1	4	0.00248
18	P10809	CH60	41.01	38.22	29.84	16.40	14	14	11	6	4	0.00224
19	Q9Y657	SPIN1	15.27	15.27	15.27	10.31	2	2	2	1	4	0.00216
20	P05141	ADT2	17.11	17.11	8.39	12.08	2	2	1	2	4	0.00182

B Most abundant exclusive interaction partners of WHx7-HA

No.	UniProt	Protein	Coverage				Unique peptides				Replicates (x/4)	Spectral Index
1	Q9Y2T2	AP3M1	3.35	3.35	0.00	0.00	1.00	1.00	0.00	0.00	2	0.00014
2	H0YK49	ETFA	5.68	6.67	0.00	0.00	1.00	1.00	0.00	0.00	2	0.00014
3	C9IYQ7	NUP35	16.52	16.52	0.00	0.00	1.00	1.00	0.00	0.00	2	0.00014

Suppl. Table 4: Interaction partners of WHV WHx7

A Most abundant interaction partners of WHx8-HA

No.	UniProt	Protein	Coverage				Unique peptides				Replicates (x/4)	Spectral Index
1	Q13885	TBB2A	40.00	29.66	26.29	26.29	1	1	1	1	4	0.04050
2	P04350	TBB4A	36.26	34.23	33.78	0.00	1	1	2	0	3	0.03212
3	Q16531	DDB1	20.00	15.26	11.93	15.18	20	13	9	14	4	0.02966
4	P07437	TBB5	42.12	38.50	37.09	34.98	3	3	3	3	4	0.02964
5	P68371	TBB4B	42.02	36.85	35.51	33.48	1	1	1	3	4	0.02847
6	Q9BVA1	TBB2B	40.00	29.66	26.29	0.00	1	1	1	0	3	0.02139
7	P68363	TBA1B	0.00	37.69	37.47	31.49	0	2	2	1	3	0.01980
8	Q9BQE3	TBA1C	39.20	35.63	35.41	31.18	2	1	1	1	4	0.01907
9	P08670	VIME	36.70	39.06	36.27	12.06	14	14	15	4	4	0.01550
10	Q9BUF5	TBB6	23.54	20.40	17.94	17.94	5	2	2	2	4	0.01298
11	P62805	H4	33.98	33.01	33.01	0.00	4	3	3	0	3	0.00443
12	Q96KK5	H2A1H	21.88	21.88	21.88	21.88	1	1	1	2	4	0.00443
13		WHx7-HA	30.00	23.33	30.00	23.33	4	3	4	3	4	0.00419
14	O60814	H2B1K	19.84	11.90	19.84	0.00	1	1	2	0	3	0.00395
15	Q9Y657	SPIN1	25.57	15.27	20.23	3.82	3	2	3	1	4	0.00393
16	G3V576	HNRPC	33.97	25.11	29.39	0.00	8	5	6	0	3	0.00387
17	P10809	CH60	36.30	23.91	25.13	20.94	13	10	9	7	4	0.00336
18	P52272	HNRPM	23.97	13.01	18.36	4.79	7	5	6	2	4	0.00257
19	Q5JUX0	SPIN3	20.93	15.50	19.38	5.04	2	2	3	1	4	0.00256
20	O14654	IRS4	11.77	9.71	8.83	3.34	8	7	6	2	4	0.00251

B Most abundant exclusive interaction partners of WHx8-HA

No.	UniProt	Protein	Coverage				Unique peptides				Replicates (x/4)	Spectral Index
1	B7Z909	HYOU1	2.86	1.71	0.00	0.00	1	1	0	0	2	0.00014
2	H0YDK8	PUM1	0.00	1.11	0.00	1.11	0	1	0	1	2	0.00014

Suppl. Table 5: Interaction partners of WHV WHx8

A Most abundant interaction partners shared by pM27-HA, pE27-HA, pR27-HA, and pUL27-HA

No.	UniProt	Protein	Spectral Index M27-HA	E27-HA	R27-HA	UL27-HA	Median Spectral Index
1	Q13885	TBB2A	0.02480	0.02264	0.03276	0.02790	0.02635
2	Q16531	DDB1	0.04931	0.06860	0.00165	0.00051	0.02548
3	P07437	TBB5	0.00785	0.01388	0.01997	0.01378	0.01383
4	P68371	TBB4B	0.00594	0.01086	0.01753	0.01214	0.01150
5	P04350	TBB4A	0.00460	0.00654	0.00893	0.01888	0.00774
6	Q9NVI7	ATD3A	0.00706	0.00578	0.01079	0.00640	0.00673
7	Q9BUF5	TBB6	0.00398	0.00023	0.00616	0.00690	0.00507
8	P78371	TCPB	0.00377	0.00598	0.00761	0.00088	0.00488
9	P52272	HNRPM	0.00319	0.00030	0.00700	0.00472	0.00395
10	O14654	IRS4	0.00401	0.00356	0.00469	0.00316	0.00378
11	Q13263	TIF1B	0.00173	0.00100	0.00491	0.00472	0.00322
12	E9PCY7	HNRH1	0.00324	0.00304	0.00368	0.00253	0.00314
13	Q14257	RCN2	0.00212	0.00272	0.00336	0.01180	0.00304
14	P17987	TCPA	0.00242	0.00355	0.00513	0.00009	0.00298
15	P68363	TBA1B	0.00270	0.00319	0.00666	0.00275	0.00297
16	Q9BQE3	TBA1C	0.00299	0.00280	0.00731	0.00284	0.00291
17	Q96TA2	YMEL1	0.00258	0.00268	0.00305	0.00244	0.00263
18	P50990	TCPQ	0.00139	0.00385	0.00528	0.00000	0.00262
19	O43823	AKAP8	0.00303	0.00160	0.00304	0.00174	0.00238
20	Q9UJS0	CMC2	0.00210	0.00182	0.00270	0.00247	0.00229

B Most abundant exclusive interaction partners shared by pM27-HA, pE27-HA, pR27-HA, and pUL27-HA

No.	UniProt	Protein	Spectral Index M27-HA	E27-HA	R27-HA	UL27-HA	Median Spectral Index
1	Q9NQH7	XPP3	0.00038	0.00036	0.00056	0.00053	0.00045

Suppl. Table 6: Shared interaction partners of pM27, pE27, pR27, and pUL27

A Most abundant interaction partners shared by HBx-HA, WHx7-HA, and WHx8-HA

No.	UniProt	Protein	Spectral Index HBx-HA	WHx7-HA	WHx8-HA	Median Spectral Index
1	Q13885	TBB2A	0.04067	0.04420	0.04050	0.04067
2	P04350	TBB4A	0.02687	0.03737	0.03212	0.03212
3	P07437	TBB5	0.02906	0.03425	0.02964	0.02964
4	P68371	TBB4B	0.02717	0.02805	0.02847	0.02805
5	Q9BVA1	TBB2B	0.02463	0.02821	0.02139	0.02463
6	Q9BQE3	TBA1C	0.02240	0.02226	0.01907	0.02226
7	P68363	TBA1B	0.00258	0.02266	0.01980	0.01980
8	Q16531	DDB1	0.00079	0.01543	0.02966	0.01543
9	Q9BUF5	TBB6	0.01240	0.01276	0.01298	0.01276
10	P08670	VIME	0.00267	0.01136	0.01550	0.01136
11	P10809	CH60	0.01047	0.00224	0.00336	0.00336
12	O14654	IRS4	0.00293	0.00299	0.00251	0.00293
13	O60814	H2B1K	0.00163	0.00286	0.00395	0.00286
14	G3V576	HNRPC	0.00194	0.00271	0.00387	0.00271
15	P62805	H4	0.00011	0.00259	0.00443	0.00259
16	P52272	HNRPM	0.00287	0.00138	0.00257	0.00257
17	Q96KK5	H2A1H	0.00150	0.00248	0.00443	0.00248
18	P08107	HSP71	0.00249	0.00093	0.00229	0.00229
19	Q9Y657	SPIN1	0.00139	0.00216	0.00393	0.00216
20	Q5JUX0	SPIN3	0.00150	0.00179	0.00256	0.00179

B Most abundant exclusive interaction partners shared by HBx-HA, WHx7-HA, and WHx8-HA

No.	UniProt	Protein	Spectral Index HBx-HA	WHx7-HA	WHx8-HA	Median Spectral Index
1	Q5JUX0	SPIN3	0.00150	0.00179	0.00256	0.00179
2	A2A2G5	GGT7	0.00042	0.00016	0.00035	0.00035
3	Q5JZB8	SPIN2B	0.00015	0.00028	0.00046	0.00028

Suppl. Table 7: Shared interaction partners of HBx, WHx7, and WHx8

A Most abundant interaction partners shared by Vpr-Flag and Vpx-Flag

No.	UniProt	Protein	Spectral Index Vpr-Flag	Spectral Index Vpx-Flag	Median Spectral Index
1	P04264	K2C1	0.03741	0.05252	0.04497
2	P13645	K1C10	0.03322	0.02210	0.02766
3	P35527	K1C9	0.00058	0.04422	0.02240
4	P01617	KVD28	0.01062	0.01351	0.01206
5	Q13885	TBB2A	0.00698	0.01150	0.00924
6	P02533	K1C14	0.00346	0.00630	0.00488
7	P08779	K1C16	0.00297	0.00357	0.00327
8	Q04695	K1C17	0.00163	0.00298	0.00230
9	O00571	DDX3X	0.00129	0.00172	0.00151
10	P32455	GBP1	0.00129	0.00107	0.00118
11	P14923	PLAK	0.00017	0.00154	0.00086
12	P35637	FUS	0.00052	0.00086	0.00069
13	H0YH81	ATP5B	0.00052	0.00080	0.00066
14	Q9UHX1	PUF60	0.00039	0.00080	0.00060
15	P05141	ADT2	0.00016	0.00093	0.00055
16	P62701	RS4X	0.00052	0.00043	0.00047
17	P31689	DNJA1	0.00039	0.00054	0.00046
18	B4DEM7	TCPQ	0.00068	0.00018	0.00043
19	Q96P63	SPB12	0.00039	0.00043	0.00041
20	O43242	PSMD3	0.00026	0.00054	0.00040

Suppl. Table 8: Shared interaction partners of Vpr and Vpx

A Most abundant interaction partners shared by pM27-HA, pE27-HA, WHx7-HA, and WHx8-HA

No.	UniProt	Protein	Spectral Index M27-HA	Spectral Index E27-HA	Spectral Index WHx7-HA	Spectral Index WHx8-HA	Median Spectral Index
1	Q16531	DDB1	0.04931	0.06860	0.01543	0.02966	0.03949
2	Q13885	TBB2A	0.02480	0.02264	0.04420	0.04050	0.03265
3	P07437	TBB5	0.00785	0.01388	0.03425	0.02964	0.02176
4	P68371	TBB4B	0.00594	0.01086	0.02805	0.02847	0.01945
5	P04350	TBB4A	0.00460	0.00654	0.03737	0.03212	0.01933
6	P68363	TBA1B	0.00270	0.00319	0.02266	0.01980	0.01149
7	Q9BQE3	TBA1C	0.00299	0.00280	0.02226	0.01907	0.01103
8	Q9BUF5	TBB6	0.00398	0.00023	0.01276	0.01298	0.00837
9	K4DI93	CUL4B	0.00845	0.00875	0.00179	0.00167	0.00512
10	Q9NVI7	ATD3A	0.00706	0.00578	0.00116	0.00038	0.00347
11	O14654	IRS4	0.00401	0.00356	0.00299	0.00251	0.00328
12	Q13619	CUL4A	0.00544	0.00553	0.00033	0.00038	0.00291
13	E9PCY7	HNRH1	0.00324	0.00304	0.00158	0.00147	0.00231
14	G3V576	HNRPC	0.00162	0.00071	0.00271	0.00387	0.00216
15	P52272	HNRPM	0.00319	0.00030	0.00138	0.00257	0.00197
16	P31689	DNJA1	0.00202	0.00016	0.00251	0.00141	0.00171
17	Q14257	RCN2	0.00212	0.00272	0.00107	0.00081	0.00159
18	P05141	ADT2	0.00166	0.00132	0.00182	0.00137	0.00152
19	Q9Y5V3	MAGD1	0.00162	0.00054	0.00162	0.00134	0.00148
20	P17987	TCPA	0.00242	0.00355	0.00049	0.00017	0.00145

B Most abundant exclusive interaction partners shared by pM27-HA, pE27-HA, WHx7-HA, and WHx8-HA

No.	UniProt	Protein	Spectral Index M27-HA	Spectral Index E27-HA	Spectral Index WHx7-HA	Spectral Index WHx8-HA	Median Spectral Index
1	Q13619	CUL4A	0.00544	0.00553	0.00033	0.00038	0.00291

Suppl. Table 9: Shared interaction partners of pM27, pE27, WHx7, and WHx8

A Most abundant interaction partners of Vpx-Flag

No.	UniProt	Protein	Coverage				Unique peptides				Replicates (x/4)	Spectral Index
1	P04264	K2C1	30.90	22.20	29.35	12.58	12	10	13	8	4	0.05252
2	P35527	K1C9	52.65	35.63	28.89	22.79	18	11	9	6	4	0.04422
3	P11021	GRP78	36.24	31.19	29.51	16.67	17	14	13	8	4	0.03481
4	P13645	K1C10	41.10	31.34	25.34	15.92	18	12	11	6	4	0.02210
5	P01617	KVD28	11.50	11.50	11.50	11.50	1	1	1	1	4	0.01351
6	Q13885	TBB2A	29.89	23.15	0.00	0.00	1	1	0	0	2	0.01150
7	P02533	K1C14	35.17	25.21	22.88	6.99	9	5	4	2	4	0.00630
8	Q9BY77	PDIP3	29.45	28.03	3.33	3.33	7	7	1	1	4	0.00543
9	P08779	K1C16	36.79	17.76	19.03	0.00	9	3	3	0	3	0.00357
10	Q9BUF5	TBB6	17.49	17.49	0.00	0.00	2	2	0	0	2	0.00348
11	Q2TAM5	p65	12.47	14.85	13.51	0.00	3	4	2	0	3	0.00314
12	Q04695	K1C17	21.76	9.95	9.95	0.00	1	1	2	0	3	0.00298
13	P68363	TBA1B	31.93	28.38	18.63	10.20	1	1	5	3	4	0.00229
14	Q9Y5V3	MAGD1	4.24	10.03	2.57	0.00	2	4	2	0	3	0.00200
15	O43852	CALU	18.10	7.62	3.49	0.00	4	2	1	0	3	0.00194
16	K7EPT6	RBP56	12.69	0.00	3.12	3.12	1	0	1	1	3	0.00192
17	O00571	DDX3X	18.43	6.95	0.00	0.00	8	3	0	0	2	0.00172
18	Q00839	HNRPU	6.67	1.45	0.00	2.18	4	1	0	1	3	0.00170
19	P22695	QCR2	11.48	8.50	3.88	0.00	3	2	1	0	3	0.00167
20	F5GWP8	PLAK	15.91	7.28	0.00	0.00	1	1	0	0	2	0.00154

B Most abundant exclusive interaction partners of Vpx-Flag

No.	UniProt	Protein	Coverage				Unique peptides				Replicates (x/4)	Spectral Index
1	Q2TAM5	p65	12.47	14.85	13.51	0.00	3	4	2	0	3	0.00314
2		Vpx-Flag	6.67	6.67	0.00	0.00	1	1	0	0	2	0.00043
3	E9PPH4	CK074	19.28	19.28	0.00	0.00	1	1	0	0	2	0.00027
4	B7Z817	DHC24	1.89	1.89	0.00	0.00	1	1	0	0	2	0.00027

Suppl. Table 10: Interaction partners of HIV-2 Vpx
